# A Green-to-Near-Infrared Photoswitch Based on a Blended Subporphyrazine–Dithienylethene System

**DOI:** 10.1021/acs.orglett.3c04320

**Published:** 2024-01-18

**Authors:** Elena Cañizares-Espada, Gema Pérez de Bustos, Koji Naoda, Atsuhiro Osuka, Tomás Torres, M. Salomé Rodríguez-Morgade

**Affiliations:** ‡ Departamento de Química Orgánica, Universidad Autónoma de Madrid, Cantoblanco, 28049 Madrid, Spain; ∥ Institute for Advanced Research in Chemical Sciences (IAdChem), Universidad Autónoma de Madrid, Cantoblanco, 28049 Madrid, Spain; ⊥ IMDEA-Nanociencia, c/Faraday 9, Cantoblanco, 28049 Madrid, Spain; # Key Laboratory of the Assembly and Application of Organic Functional Molecules of Hunan Province, Hunan Normal University, Changsha 410081, China; § Department of Chemistry, Graduate School of Science, Kyoto University, 606-8502 Kyoto, Japan

## Abstract

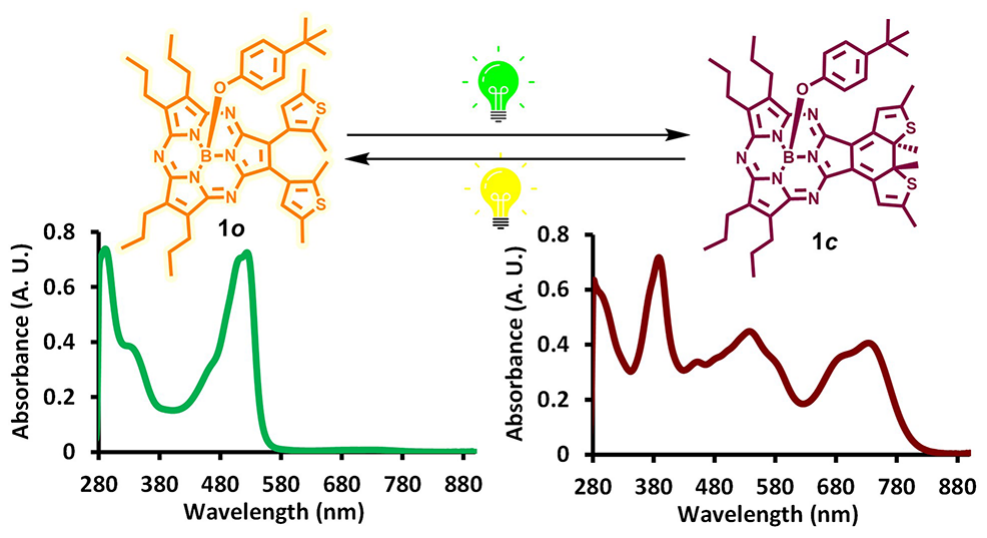

A subporphyrazine (SubPz)–dithienylethene (DTE) photochromic device with **1o** and **1c** states, was developed and characterized. In this device, the DTE unit can reversibly switch the SubPz absorbance from green to near-infrared [λ_max_ (o/c) = 527 nm/740 nm], as well as the SubPz fluorescence and singlet oxygen quantum yields. The core of this design involves using a highly tunable SubPz chromophore that shares its quasi-isolated ethene moiety with a DTE photoswitch.

Photoswitches involving activation and deactivation of biomolecules are emerging as valuable tools for biotechnological applications.^1^ In this respect, while ultraviolet (UV) light is injurious and has a shallow penetration depth through biological tissues, polymers, or gels, traditional photoswitches require high-energy, UV light irradiation to activate the photoswitching process in one direction or both directions. Therefore, there is a growing need for the development of biocompatible photoswitches, with lower-energy absorbance bands that enable activation using benign, visible, and near-infrared (NIR) light.^2^ We report here a photoswitch that exhibits green-to-near-infrared absorbance in its two states. The device consists of an intimately fused subporphyrazine (SubPz)–dithienylethene (DTE) system **1** (Scheme 1), in which the DTE can reversibly switch near-infrared SubPz absorbance [λ_max_ (o/c) = 527 nm/740 nm] and modulate its luminescence and singlet oxygen quantum yields.

**Scheme 1:**
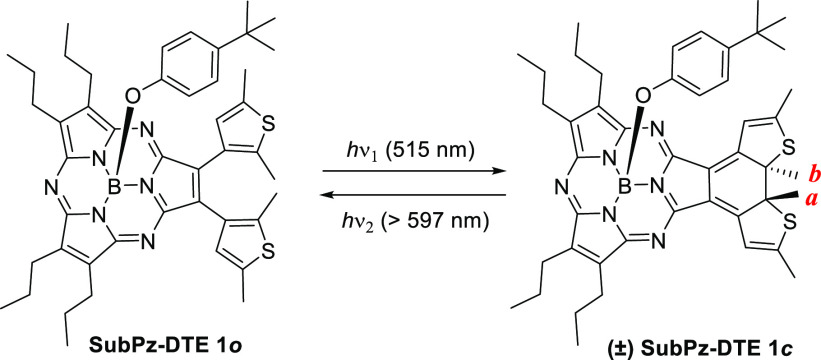
Photochromic Reaction of SubPz–DTE **1**

The SubPz component^3^ of **1** belongs to a class of curved, 14-π-electron, aromatic porphyrinoids,^4^ consisting of three pyrrole subunits connected through their 2,5-positions by aza-bridges and coordinating boron(III). The optical spectrum of the bare SubPz structure is characterized by an intense absorption at 500 nm (Q-band), accompanied by a Soret band at ∼300 nm.^5^ Importantly, SubPzs show exceptional electronic tunability via peripheral functionalization. This arises from an unusually strong influence of peripheral substituents on the SubPz π-system, owing, inter alia, to their direct attachment to the β-positions of the SubPz pyrrole rings. Thus, the absorption, luminescent, and redox profiles of SubPzs can be finely or significantly modified by peripheral functionalization with heteroatoms,^3,6^ arylation,^7−10^ or vinylation.^8^ This versatility has been used to shift the absorption of the curved SubPz chromophores to the red by ≤140 nm,^8^ design non-fullerene electron acceptors,^11^ and construct a SubPz–pentacene conjugate that undergoes enhanced intramolecular singlet fission (i-SF).^12^

SubPzs lacking fused rings at their periphery contain a quasi-isolated C_β_=C_β_ bond that is not included in the 14-π-electron circuit. We have combined the reactivity of this “ethene” moiety with the unique tunability of SubPzs to design a DTE photochromic switch,^13^ which exists in two photochemically active states, i.e., the open and closed states. A key feature of this design is that the two chromophores, i.e., SubPz and DTE, share the ethene unit involved in the DTE electrocyclic ring closure. Consequently, we anticipated a closed state in **1** with a deeply perturbed SubPz π-system, which would lead to red-shifted absorption and altered luminescent properties.

SubPz–DTE **1** consists of a A_2_B type SubPz,^14^ in which the B unit is endowed with two 2,5-dimethylthiophene rings that are directly connected to the macrocycle (Scheme 1). A units and the axial position are substituted with alkyl substituents and a *tert*-butylphenoxy group, respectively, both providing solubility and stability to the system, without altering the absorption profile.^12,15,16^ Hybrid **1** was assembled in 88% yield by a Liebeskind–Srogl coupling of bis-thioether-substituted SubPz **4**, with 2,5-dimethyl-3-boronic acid (**5**),^17^ following the previously reported methodology for peripheral functionalization of SubPzs (Scheme 2).^7,8^

**Scheme 2:**
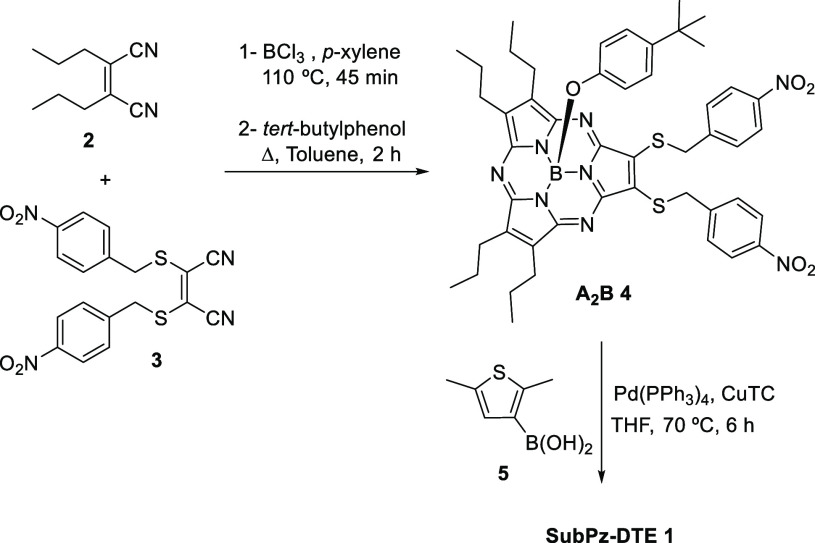
Synthesis of SubPz–DTE Photoswitch **1**

SubPz intermediate **4** was prepared by crossover cyclotrimerization of dipropylmaleonitrile (**2**) and bis-*p-*nitrobenzylsulfanyl maleonitrile (**3**) (Scheme 2). This type of mixed cyclization is by far the most common procedure for preparing unsymmetric azaporphyrin derivatives,^14^ although this work constitutes the first example within SubPzs. The reaction gave rise to a mixture of two SubPzs, namely, the symmetric hexapropyl-SubPz (A_3_) and the unsymmetric (A_2_B) SubPz **4**, which were separated by chromatography. Here, the choice of nitrobenzyl substitution for maleonitrile **3** is crucial, as the polarity of this moiety is higher than that of the propyl groups, thus enabling the chromatographic separation of the two A_3_ and A_2_B SubPzs. In fact, using the typical thioalkyl substitution^7,8^ led to inseparable mixtures of SubPzs.

The ^1^H NMR spectrum of SubPz–DTE **1** in toluene (Figure S16) showed the typical signals corresponding to the axial *tert*-butylphenoxy ligand as one singlet at 1.03 ppm and two doublets at 6.72 and 5.50 ppm, very much shielded owing to the SubPz diatropicity. The desymmetrization of the molecule on going from the A_3_^3a^ to A_2_B system **1** is clearly evidenced by the resonances corresponding to the peripheral propyl groups, which exhibit three multiplets between 2.8 and 3.2 ppm assigned to the methylene groups directly attached to the SubPz ring. A signal at 6.91 ppm accounts for the thiophene aromatic proton. Unlike other examples of DTE systems containing dimethylthiophene and phenanthroline units,^18^ the ^1^H NMR spectrum of **1** in the open state (**1o**) indicates unrestricted rotation of the two thiophene rings, displaying two singlets at 2.49 and 2.14 ppm corresponding to the 2- and 5-methyl substituents, respectively. The UV–visible (UV–vis) absorption spectrum also attests to the low symmetry of SubPz **1**, revealing in toluene a split Q-band with maxima at 508 and 527 nm (Figure 1 and Figure S18), strongly red-shifted with respect to the isolated DTE unit [λ_max_ (o/c) = 270 nm/220 nm],^19,20^ accompanied by a shoulder at 465 nm. Two more absorptions at 291 and 329 nm correspond to the Soret band. In addition, the fluorescence spectrum (Figure S20) shows a maximum at 568 nm (Stokes shift of 1369 cm^–1^) that mirror images the Q-band, with a fluorescence quantum yield Φ_F_ of 0.05.

**Figure 1:**
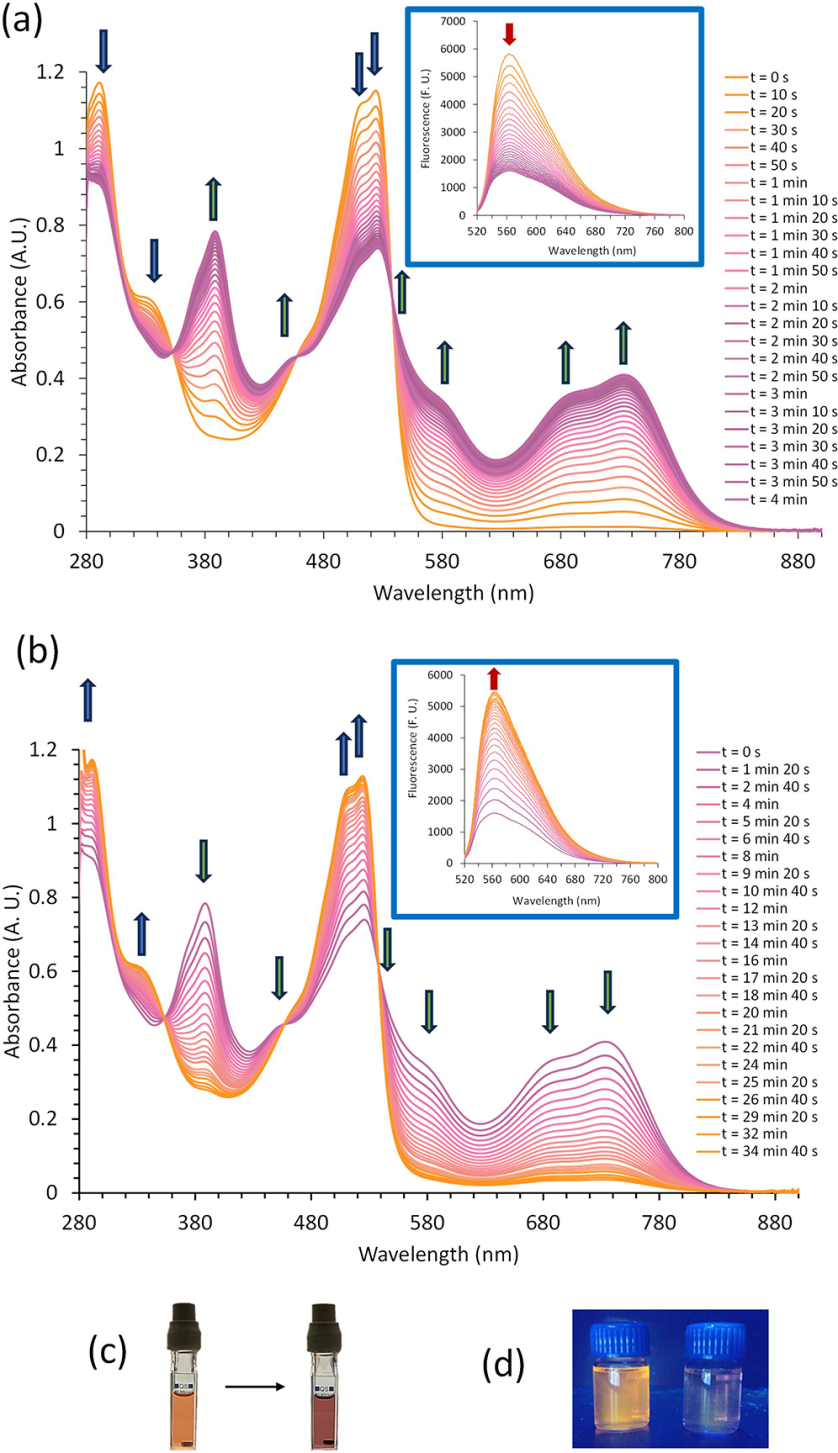
UV–vis and fluorescence spectroscopies of a toluene solution of SubPz–DTE **1** (2.34 × 10^–5^ M, 298 K). (a) Irradiation with 515 nm light until the photostationary state is reached. (b) Irradiation with 597 nm light until the open state is recovered. (c) Color change in the solution upon irradiation with 515 nm light. (d) Change in the fluorescence of the solution upon irradiation with 515 nm light.

The photochromic behavior of SubPz–DTE **1** was studied by irradiation in toluene with 515 and 597 nm LED lights and monitored by UV–vis, fluorescence, and ^1^H NMR spectroscopies. Upon irradiation with 515 nm light, the orange solution of **1o** turned pink-violet (Figure 1c).

The intensities of the absorption bands at 291, 329, 508, and 527 nm decreased, while new bands at 388, 452, 538, 585, 687, and 740 nm that characterize **1c** emerged (Figure 1a). Three isosbestic points at 349, 471, and 536 nm indicate the existence of the two open and closed interconverting isomers of **1** and exclude the presence of any intermediate or other simultaneous photochemical processes. A photostationary state (PSS) was reached after irradiation for 4.5 min.

Irradiation of **1o** in toluene at −7 °C with 515 nm light did not shift the PSS to higher conversion into **1c**, thus discarding a possible ring-opening thermal process hindering the advance of the photoisomerization of **1o** into **1c**. Indeed, the photostationary state mixture in the solid state was stable at room temperature in the dark, while in solution, it reverted to **1o** within weeks. Complete back isomerization could be thermally induced in 45 min upon heating in toluene at 80 °C (Figure S27).

The changes produced in the optical spectrum of **1o** upon conversion into **1c** are far more striking than those observed for other photochromic devices consisting of a DTE-substituted porphyrin^21,22^ or in a SubPc axially substituted with a DTE unit.^23^ The λ_max_ shift upon photoisomerization is also much larger than that produced in a related bis-DTE-porphyrazine hybrid [λ_max_ (o/c) = 650 nm/711 nm]^24^ and in a porphyrazine dimer bearing two opposite, switchable DTE units [λ_max_ (o/c) = 745 nm/795 nm],^25^ evidencing the influence of the electronically deficient boron atom in the perturbation of the SubPz π-system by peripheral substituents.^8,26^ In fact, the absorption at 740 nm of **1c** essentially corresponds to a π → π* transition in the highly delocalized SubPz–DTE system, as predicted by TD-DFT-B3LYP/6-31G(d,p) calculations. The predicted frontier orbitals and the absorption bands (λ_max_) with the dominant electronic transitions for SubPz–DTE **1o** and **1c** are included in Figures S29 and S30 and Table S1, respectively.

The photoisomerization of SubPz–DTE **1** was also monitored by fluorescence spectroscopy (λ_ex_ = 512 nm). Upon irradiation with 515 nm light, the intensity of the fluorescence band decreased by ≤27% and the band slightly shifted to 560 nm in the PSS (Figure 1a, inset). No fluorescence band was observed upon excitation at 730 nm, so that we could calculate a 73% conversion of **1o** into **1c** in the PSS. By subtracting the initial absorption spectrum of **1o** (27% of the initial intensity) from the absorption spectrum of the PSS mixture, we could estimate the UV–vis spectrum of **1c** (Figure S25). Fluorescence quenching on going from **1o** to **1c** can also be seen with the naked eye (Figure 1d) under 254 nm UV light. These results suggest that the closed DTE form quenches the SubPz S_1_ excited state. The fluorescence quenching could be attributed to intramolecular electron^21^ or energy transfer processes^25^ between the SubPz and dihydrothiophene units, similar to processes within porphyrin and DTE.^22^ Alternatively, the deactivation of SubPz S_1_ in **1c** could occur by intersystem crossing leading to the lower-energy T_1_ state. This last hypothesis is supported by the higher values of singlet oxygen quantum yields (Φ_Δ_ = 0.7) estimated for the mixture of **1o** and **1c** obtained in the PSS, related to the values obtained for the open **1o** photoisomer (Φ_Δ_ = 0.4) (see the Supporting Information).

Irradiation of the solution of **1** in the PSS with 597 nm light caused the absorption bands of **1c** in the near-infrared region to disappear with concomitant recovery of the bands corresponding to **1o**, as well as its fluorescence (Figure 1b and inset). Quantum yields of Φ_OC_ = 3.43 × 10^–4^ for photocyclization and of Φ_CO_ = 6.50 × 10^–6^ for photocycloreversion were determined using a reported procedure,^27^ upon irradiation of **1o** with 515 nm and **1c** with 597 nm LED lights. The interconversion between **1o** and **1c** could be repeated up to 24 times without obvious light fatigue (Figure 2) or important signs of degradation (Figure S28), pointing to the excellent switching properties of SubPz–DTE **1**.

**Figure 2:**
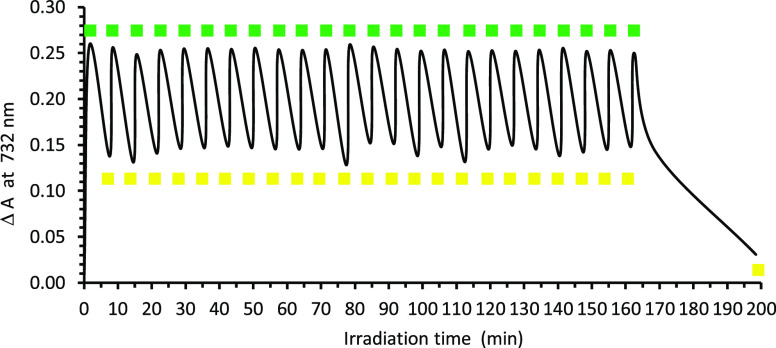
Photoswitching performance of a toluene solution of SubPz–DTE **1** (2.00 × 10^–5^ M, 298 K) monitored by UV–vis spectroscopy at 732 nm. In the first cycle, **1o** is irradiated with 515 nm light for 1.5 min, followed by irradiation with 597 nm light for 6 min. In the 23 subsequent cycles, the solution is irradiated with 515 nm light for 1 min, followed by irradiation with 597 nm light for 6 min. The last irradiation with 597 nm light was performed for 30 min, until no more photoisomerization was observed. Exposure to 515 nm light triggers isomerization to **1c** (green squares); subsequent exposure to 597 nm light isomerizes the sample back to **1o** (yellow squares).

The photoisomerization of **1** was additionally studied by ^1^H NMR spectroscopy, irradiating a solution of **1o** in deuterated toluene (6.38 × 10^–3^ M), with 515 nm light (Figure 3 and Figure S22). The PSS was reached in 80 min and consisted of a 1:2 mixture of **1o** and **1c**, a ratio that was estimated by integrating the signals corresponding to the axial *tert*-butylphenoxy groups of the two photoisomers and which matched that inferred by fluorescence spectroscopy. ^1^H NMR spectroscopy also confirmed the structure of the closed form. **1c** consists of a racemic mixture in which the methyl substituents of the thiophene ring become non-equivalent (see Scheme 1). On going from the open to the closed form, we observed four new signals assigned to four methyl protons at 2.34, 1.96, 1.76, and 1.73 ppm, while the intensity of the corresponding resonances of **1o** at 2.49 and 2.14 ppm decreased (Figure 3 and Figure S22). The splitting of these methyl signals into four resonances arises from the anisotropy originated by the SubPz convex and concave faces, while the upfield shift of the 5-methyl protons and, especially, the 2-methyl protons is typical of this type of photoisomerization.^24^ By analogy to that observed for rutheroarene π-complexes of SubPcs,^16b^ we tentatively assign the signals at 1.76 and 1.73 ppm to methyl groups a and b of **1c** (Scheme 1), pointing to the SubPz convex and concave faces, respectively, the latter slightly more shielded by the domed π-system.^16b^ On the contrary, the *tert*-butylphenoxy and thiophene aromatic protons shift to 6.00, 6.83, and 7.04 ppm, respectively. Open state **1o** was recovered upon irradiating the photoisomeric mixture with 597 nm light (Figure S23).

**Figure 3:**
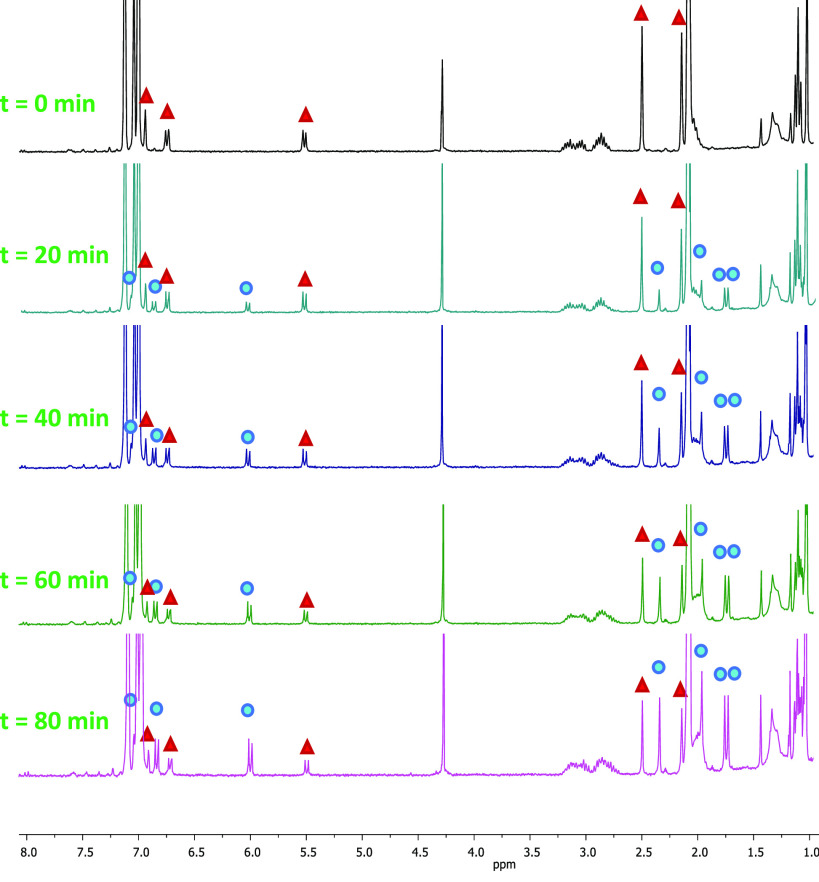
^1^H NMR spectral changes of SubPz–DTE **1o** in deuterated toluene upon photoexcitation at 515 nm. Resonances of dimethylthiophene and axial ligand are represented by red triangles for **1o** and blue circles for **1c**.

In summary, in this study, we have successfully designed a fused SubPz–DTE system, demonstrating its effectiveness as a near-infrared photoswitchable absorber that can be activated and deactivated with visible light. Furthermore, the emission and generation of singlet oxygen in this hybrid are also reversibly modulated through photoisomerization. The development of activatable singlet oxygen photosensitizers is attracting more attention because they provide additional selectivity to the already localized nature of PDT functioning.^28^ The key to our design is the use of a highly tunable subporphyrazine that shares its quasi-isolated ethene moiety with a dithienylethene photoswitch. This work lays the foundation for future developments of visible light/NIR photoswitchable systems that are compatible with biological environments and operating within the biological window, which will be reported in due course.

## Data Availability

The data underlying this study are available in the published article and its Supporting Information.

## Supporting Information Available

Experimental details, compound characterization data, and NMR, IR, MS, UV–vis, and fluorescence spectra (PDF)
DFT calculations for **1o** (MOL)
DFT calculations for **1c** (MOL)

## Supplementary Material

ol3c04320_si_001.pdf

ol3c04320_si_002.mol

ol3c04320_si_003.mol
