# Supplementary material for: A Green-to-Near-Infrared Photoswitch Based on a Blended Subporphyrazine–Dithienylethene System
Source: Org Lett. 2024 Jan 18;26(4):955–9. doi: 10.1021/acs.orglett.3c04320 (PMC10845152; doi:10.1021/acs.orglett.3c04320)
Supplement: Supplementary file 1 — ol3c04320_si_001.pdf [file ol3c04320_si_001.pdf]

SUPPORTING INFORMATION

A Green-to-Near-Infrared Photoswitch Based on a Blended Subporphyrazine-Dithienylethene System

Elena Cañizares-Espada,[†,a] Gema Pérez de Bustos,[†,a] Koji Naoda,[a,e] Atsuhiro Osuka,[d,e]* Tomás Torres,[a,b,c,]* and M. Salomé Rodríguez-Morgade[a,b]*

[a] Departamento de Química Orgánica, Universidad Autónoma de Madrid (UAM), Cantoblanco, 28049 Madrid, Spain

[b] Institute for Advanced Research in Chemical Sciences (IAdChem), Universidad Autónoma de Madrid (UAM), 28049 Madrid, Spain

[c] Instituto Madrileño de Estudios Avanzados (IMDEA)-Nanociencia, C/ Faraday, 9, Cantoblanco, 28049 Madrid, Spain

[d] Key Laboratory of the Assembly and Application of Organic Functional Molecules of Hunan Province, Hunan Normal University, Changsha 410081 (China).

[e] Department of Chemistry, Graduate School of Science, Kyoto University, 606-8502 Kyoto (Japan).

Corresponding authors:

salome.rodriguez@uam.es

tomas.torres@uam.es

atsuhiroosuka@hunnu.edu.cn

†These authors contributed equally

CONTENTS

1. General Experimental Procedures S3
2. Figure S1. Emission spectrum of the green LED of the photochemical system (λ_{max}=515 nm) S4
3. Figure S2. Emission spectrum of the yellow LED of the photochemical system (λ_{max}=597 nm) S4
4. Synthetic procedures and characterization S6
5. 2,3-bis((4-nitrobenzyl)thio)maleonitrile (**3**) S6
6. 4-*tert*-Butylphenoxy[2,3-bis-(nitrobenzylsulfanyl)-7,8,12,13-tetrapropylsubporphyrazinato] boron (III) (SubPz-A_2B **4**) S6
7. SubPz-DTE **1*o*** S7
8. SubPz-DTE **1*c*** S7
9. Spectra of **3** S9
10. Spectra of **4** S12
11. Spectra of SubPz-DTE **1*o*** S16
12. Photoisomerization of SubPz-DTE **1*o*** – **1*c*** S20
13. Figure S22. ^{1}H NMR spectral changes of SubPz-DTE 1 in deuterated toluene upon photoexcitation at 515 nm. S20
14. Figure S23. ^{1}H NMR spectral changes of the PSS of SubPz-DTE **1** in deuterated toluene upon photoexcitation at 597 nm. S21
15. Figure S24. ^{13}C NMR (75.5 MHz, toluene-d_8) of the PSS mixture of SubPz-DTE **1** S22
16. Figure S25. UV-Vis spectrum of **1*c*** estimated by subtracting the initial absorption spectrum of **1*o*** (27% of the initial intensity), from the absorption spectrum of the PSS mixture. S22
17. Figure S26. HRMS (APCI$^+$, DCM): of **1*o*** after 80 minutes of irradiation with 515 nm LED light. Isotopic pattern: Upper: found; Lower: calculated S23
18. Figure S27. Changes in the UV/vis absorption spectrum of a mixture of **1*o*** and **1*c*** in the PPS ratio in toluene upon heating at 80ºC, monitoring the thermal cycloreversion. S24
19. Figure S28. Evolution of absorbance monitored by UV-Vis spectroscopy in a toluene solution of SubPz-DTE **1** during 24 repetitive switching cycles. S24
20. Figure S29. DFT predicted HOMO and LUMO orbitals and frontier orbital energy levels (eV) for a) **1*o*** and b) **1*c***. S25
21. Figure S30. TD-DFT predicted absorption spectra for a) **1*o*** and b) **1*c***. S25
22. Table S1. Predicted and experimental absorption bands (λ_{max}), oscillator strengths (*f*) and dominant electronic transitions, for SubPz-DTE **1*o*** and **1*c*** S26
23. Figure S31. Photodecomposition of DPBF by $^1O_2$ in DMSO after irradiation of **1*o*** monitoring the maximum absorption of DPBF at 417 nm. S27
24. Figure S32. Photodecomposition of DPBF by $^1O_2$ in DMSO after irradiation of the mixture of **1*o*** and **1*c*** in the PSS ratio monitoring the maximum absorption of DPBF at 417 nm. S27
25. References S28

General Experimental Procedures.

Synthesis.

All manipulations were carried out under an argon atmosphere. Irradiations were conducted under argon in Hellma quarzglas high performance fluorescence cells (117.100-QS) with 10 mm light path, and in 5 mm diam. NMR tubes. The light sources were monochromatic SMD3528 LED lights. Column chromatographies were conducted on silica gel Merck-60 (230-400 mesh, 60 Å). TLC was performed on aluminium sheets pre-coated with silica gel 60 F_{254} (E. Merck). Chemicals were purchased from Aldrich Chemical Co., Alfa Aesar (Thermo Fisher Scientific), and TCI Europe N. V. and used as received without further purification. "Synthetic grade" solvents were used for chemical reactions, column chromatography purifications, and "anhydrous grade" for reactions under dry conditions. Additionally, some solvents were further dried by distillation with sodium/benzophenone (THF) or with solvent purifying system by Innovative Technology Inc. MD-4-PS. *Cis*-1,2-dicyano-1,2-ethylenedithiolate,[1] dipropylmaleonitrile **2**,[2] 2,5-dimethyl-3-boronic acid (**5**)[3] and CuTC[4] were prepared following reported procedures.

Instrumental Analyses.

^{1}H NMR and **^{13}C NMR** were recorded on a Bruker AC-300 (300 MHz) spectrometer using as deuterated solvent, $CDCl_3$ or toluene-d_8. The temperature was actively controlled at 298 K. Chemical shifts are measured in ppm relative to tetramethylsilane (TMS). **UV/Vis** spectra were recorded with a Jasco V-660-Spectrophotometer. **IR** spectra were recorded with Agilent Technologies Cary 630 FTIR, or using a Bruker Alpha II spectrometer by attenuated total reflection (ATR). **Mass spectra** (**MS**) were acquired by MALDI-TOF technique in SidI, using a Bruker REFLEX III with a nitrogen laser operating at 337 nm, or using Atmospheric Pressure Chemical Ionization (APCI) as ionization method and using a Q-TOF analyzer. **Fluorescence** was recorded with a JASCO-V8600-Spectrofluorometer. Fluorescence quantum yields were calculated using fluorescein as the reference (Φ_F(EtOH) =0.79).

Singlet oxygen quantum yields.

The singlet oxygen quantum yields (Φ_Δ) were measured in DMSO by monitoring, by UV-Vis spectroscopy, the photoinduced decomposition of a $^1O_2$ quencher, after irradiation of oxygen-saturated solutions of each PS with a halogen lamp. 1,3-diphenylisobenzofuran (DPBF) was used as quencher and ZnPc as the reference compound (Φ_Δ = 0.67).[5,6] Singlet oxygen quantum yields (Φ_Δ) were calculated according to the following equation:

$$\phi_\Delta^S = \phi_\Delta^R \frac{k^S I_{aT}^R}{k^R I_{aT}^S}$$

where k is the slope of a plot of ln(A_0/A_t) versus irradiation time t, with A_0 and A_t being the absorbances of the scavenger at the monitored wavelength (417 nm), before and after irradiation time t, respectively. R and S superscripts indicate the reference and sample, respectively. I_{aT} is the total amount of light absorbed by the dye and it is given by the sum of the intensities of the absorbed light I_a at wavelengths from 530 nm (DMSO) to 800 nm (step 0.5 nm). Light under 530 nm is completely filtered off. I_a at a given wavelength is calculated using Beer's law:

$$I_a = I_0(1 - e^{-2,3\,A})$$

where I_0 corresponds to the transmittance of the filter at a given wavelength and A refers to the absorbance of the dye at that wavelength.

Photocyclization and photocycloreversion reactions.

The emission spectrum of the LED sources and the intensity of the light were measured with a spectrometer equipment Stellarnet model UV-VIS-NIR. The green light was of 515 nm (10.0 W), and

the yellow light was of 597 nm (3.7 W). The emission spectra of the green LED and the yellow LED of the photochemical system are represented in Figures S1 and S2. The distance between the light source and the reaction mixture was of 3.5 cm.

Figure S1. Emission spectrum of the green LED of the photochemical system (λ_{max}=515 nm)

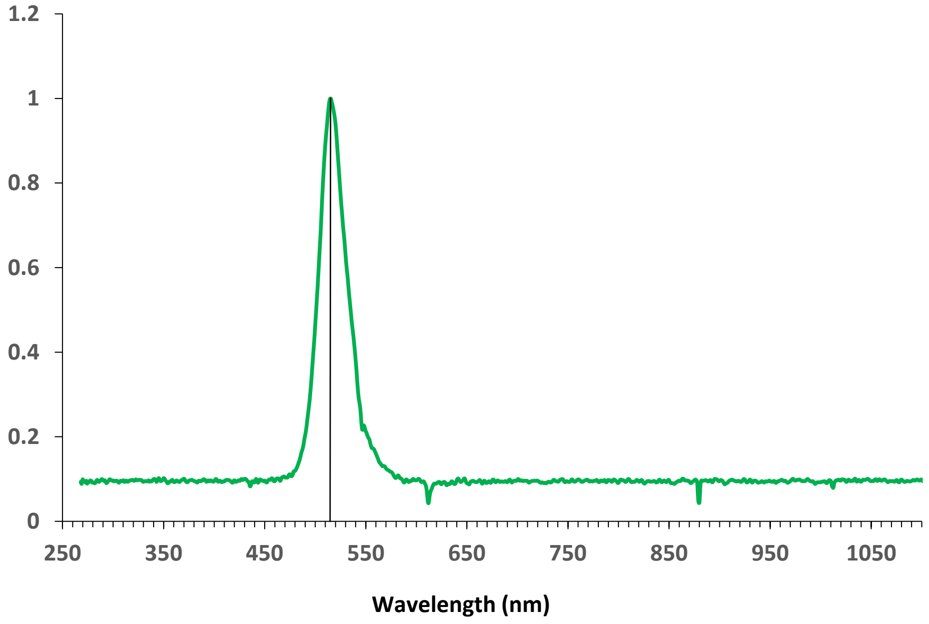

Figure S2. Emission spectrum of the yellow LED of the photochemical system (λ_{max}=597 nm)

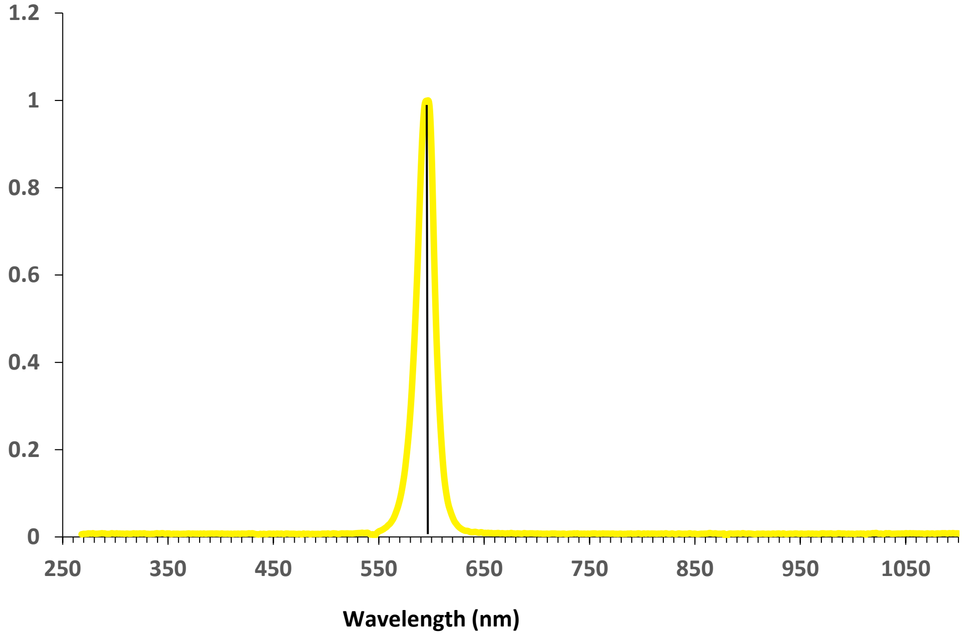

Theoretical calculations.

DFT calculations were performed using the GAUSSIAN 16, Revision C.01 package.[7] The structures of **1*o*** and **1*c*** were freely optimized *in vacuo* using the 6-31G(d,p) basis set and the CAM-B3LYP and B3LYP functionals, respectively. The same level of theory was used to compute the energies and electronic distributions of the frontier orbitals. For excited states, TD-CAM-B3LYP/6-31G(d,p) and TD-B3LYP/6-31G(d,p) calculations were performed. B3LYP functional was selected for **1*c*** because it is commonly used for SubPcs and SubPzs,[8] and the predicted absorption spectrum matched quite well with that obtained experimentally. CAM-B3LYP functional, however predicted a more accurate absorption spectrum for **1*o***.

Synthetic procedures and characterization

Synthesis of the SubPz-DTE photoswitch 1.

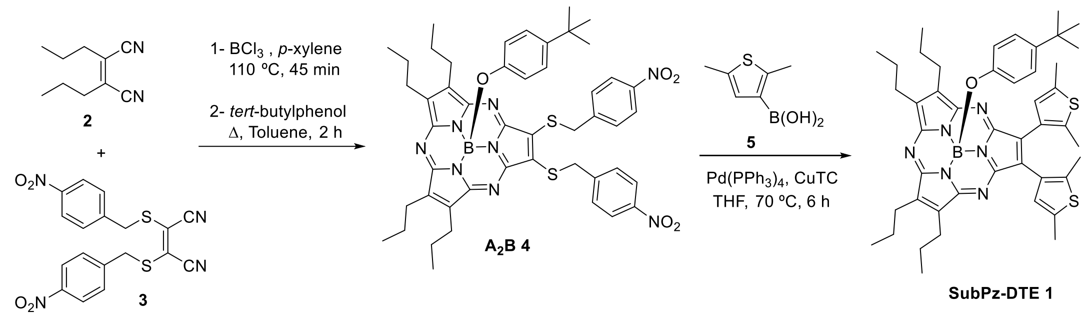

2,3-bis((4-nitrobenzyl)thio)maleonitrile (3)

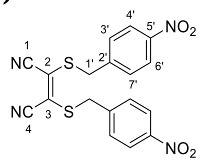

A mixture of *cis*-1,2-dicyano-1,2-ethylenedithiolate (5.0 g, 26.9 mmol), 4-nitrobenzyl chloride (9.23 g, 53.8 mmol) and NaI in acetone (400 mL) was heated to reflux in an oil bath for 48 h. The solution was rotary evaporated. The residue was taken up in hexanes and washed three times with water. The organic layer was dried with Na_2SO_4, and the solvent was removed by rotary evaporation to yield a yellow solid that was purified by column chromatography on silica gel using a (20:1) mixture of heptane/AcOEt as the eluent. Product **3** was obtained as a yellowish solid (1.42 g, 65 %).
^{1}H NMR (300 MHz, $CDCl_3$, δ ppm): 8.21 (d, *J* = 8.7 Hz, 2H), 7.52 (d, *J* = 8.7 Hz, 2H), 4.38 (s, 2H). ^{13}C-NMR (75.5 MHz, $CDCl_3$, δ ppm): 147.9, 141.8, 130.2, 124.4, 121.5, 111.9, 38.4. FT-IR (ATR) υ (cm^{-1}): 3077 ($C\text{-}H_{arom}$), 2945, 2924 (C=C-H), 2848 (CH_2), 2205 (C≡N), 1599, 1511, 1417, 1342, 1179, 1105, 1057, 1011, 866, 857, 797, 734, 701, 622, 484. HRMS (APCI) *m/z*: $[M + H]^+$ Calcd for $C_{18}H_{13}N_4O_4S_2$ 413.0373; Found 413.0367.

4-*tert*-Butylphenoxy[2,3-*bis*-(nitrobenzylsulfanyl)-7,8,12,13-tetrapropylsubporphyrazinato] boron (III) (SubPz-A_2B 4)

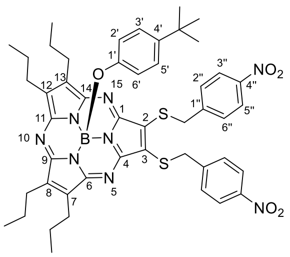

To maleonitriles **2** (141 mg, 0.87 mmol) and **3** (180 mg, 0.44 mmol) a 1.0 M solution of BCl_3 in *p*-xylene (1,3 mL, 1.3 mmol) was added under argon and the mixture was stirred at 140 ºC for 60 min in an oil bath. The excess of BCl_3 was removed with an argon stream and the solvent was rotary evaporated. A solution of 4-*tert*-butylphenol (0.98 g, 6.5 mmol) in toluene (3.0 mL) was then added to the residue and the mixture was refluxed for 2 h in an oil bath. After removing the solvent by rotary evaporation, the crude was chromatographed on silica gel using a (20:1) mixture of heptane/AcOEt as

the eluent. The third orange fraction was collected and further purified by gel permeation chromatography on Biobeads using toluene as the eluent, affording SubPz **4** (11.3 mg, 3.0 %).
^{1}H-NMR (300 MHz, $CDCl_3$, δ ppm): 8.06 (d, *J* = 8.3 Hz, 2H), 7.61 (d, *J* = 8.5 Hz, 4H), 6.81 (d, *J* = 8.5 Hz, 2H), 5.26 (d, *J* = 13.4 Hz, 2H), 5.16 (d, *J* = 8.4 Hz, 2H), 4.90 (d, *J* = 13.3 Hz, 2H), 3.2-2.9 (m, 8H), 2.16-1.98 (m, 8H), 1.23 (t, *J* = 7.4 Hz, 12H), 1.14 (s, 9H). ^{13}C-NMR (75.5 MHz, $CDCl_3$, δ ppm): 159.4, 157.5, 154.6, 147.7, 146.4, 144.4, 138.0, 137.7, 130.5, 126.0, 124.1, 118.7, 115.2, 39.3, 34.3, 31.6, 27.3, 27.2, 25.4, 25.2, 14.8, 14.7. UV-Vis ($CHCl_3$, nm): λ_{max} (log ε/dm^3 mol^{-1} cm^{-1}): 278 (4.9), 349 (4.5), 463 (4.3), 517 (4.6), 531 (4.7). FT-IR (ATR) υ (cm^{-1}): 2958 (C=C-H), 2868 (CH_3, CH_2), 1457, 1360, 1237, 1176, 1019. Fluorescence ($CHCl_3$, nm) λ_{ex} = 516; λ_{em} = 572. MS (MALDI-TOF$^+$, DCTB+NaI): *m/z* = 918.5-923.5 [M + Na]$^+$. HRMS **(**MALDI-TOF) *m/z*: [M + Na]$^+$ Calcd for $C_{48}H_{53}BN_8O_5S_2Na$ 918.3602; Found: 918.3587.

SubPz-DTE 1*o*

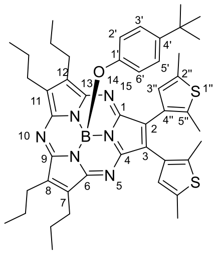

To SubPz **4** (20 mg, 31 µmol), $Pd(PPh_3)_4$ (8.1 mg, 30 mol %), CuTC (43.0 mg, 0.23 mmol), boronic acid **5** (40.0 mg, 0.26 mmol), and anhydrous THF (3.0 mL) were added, and the mixture was refluxed for 6 h in an oil bath. The solvent was rotary evaporated, and the residue chromatographed on silica gel using a (20:1) mixture of heptane/AcOEt as the eluent. SubPz-DTE **1** was collected as the red fraction (15 mg, 88 %).
^{1}H-NMR (300 MHz, $CDCl_3$, δ ppm): 6.75 (d, *J* = 8.7 Hz, 2H), 6.54 (q, *J* = 1.0 Hz, 2H), 5.21 (d, *J* = 11.8 Hz, 2H), 3.2-2.9 (m, 8H), 2.44 (s, 6H), 2.39 (s, 6H), 2.02 (m, 8H), 1.16 (t, *J* = 7.12 Hz, 12H), 1.15 (s, 9H). ^{1}H-NMR (300 MHz, toluene-d$_8$, δ ppm): 6.91 (s, 2H), 6.72 (d, *J* = 7.7 Hz, 2H), 5.50 (d, *J* = 7.8 Hz, 2H), 3.2 - 2.8 (m, 8H), 2.49 (s, 6H), 2.14 (s, 6H), 2.1-2.0 (m, 8H), 1.11 (t, *J* = 7.4 Hz, 12H), 1.03 (s, 9H). ^{13}C-NMR (75.5 MHz, toluene-d$_8$, δ ppm): 158.7, 157.1, 155.7, 152.3, 143.6, 137.9, 136.1, 135.6, 134.6, 133.1, 130.4, 124.0, 119.4, 34.7, 34.2, 31.9, 30.8, 27.4, 25.6, 25.5, 15.4, 15.0, 14.3. UV-Vis (Toluene, nm): λ_{max} (log ε/dm^3 mol^{-1} cm^{-1}): 291 (4.9), 329 (4.6), 464 (sh), 508 (sh) 527 (4.9). FT-IR (ATR) υ (cm^{-1}): 2956 (C=C-H), 2853 (CH_3, CH_2), 1726, 1670, 1457, 1245, 1016, 835, 725. Fluorescence (Toluene, nm) λ_{ex} = 510; λ_{em} = 565. HRMS (APCI$^+$) *m/z*: [M + H]$^+$ Calcd for $C_{46}H_{56}BN_6OS_2$ 782.4081; Found 782.4054.

SubPz-DTE 1*c*

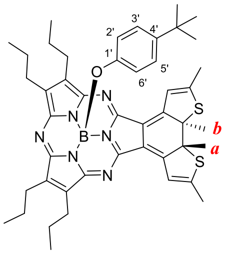

In an NMR tube, a solution of **1*o*** (4 mg, 5.11 µmol) in toluene-d$_8$, was irradiated for 80 min with 515 nm LED light and the changes in the ^{1}H NMR spectrum were recorded until a PSS was achieved.

Integration of the signals corresponding to the *tert*-butylphenoxy group revealed a 1:2 ratio for **1*o*** and **1*c***, respectively. **1*c*** was characterized from this mixture. The UV-Vis spectrum of **1*c*** was estimated by subtracting the initial absorption spectrum of **1*o*** (27% of the initial intensity), from the absorption spectrum of the PSS mixture, considering a 73% conversion of **1*o*** into **1*c*** in the PSS (calculated by fluorescence spectroscopy). ^{1}H-NMR (300 MHz, toluene-d_8, δ ppm): 7.16 (s, 2H), 6.95 (d, *J* = 8.5 Hz, 2H), 6.12 (d, *J* = 8.6 Hz, 2H), 3.1-2.8 (m, 8H), 2.46 (s, 3H), 2.15-2.12 (m, 8H), 2.08 (s, 3H), 1.88 (s, 3H), 1.85 (s, 3H), 1.23 (t, *J* = 5.6 Hz, 12H), 1.16 (s, 9H). ^{13}C-NMR (75.5 MHz, toluene-d_8, δ ppm): 158.7, 157.5, 157.2, 157.1, 155.7, 152.3, 150.2, 150.0, 143.6, 143.3, 137.9, 136.3, 136.1, 135.6, 134.6, 133.1, 130.4, 124.0, 119.7, 119.4, 79.2, 78.1, 70.6, 68.9, 34.7, 34.4, 34.2, 31.9, 30.8, 27.4, 27.2, 27.1, 26.4, 25.6, 25.5, 18.3, 15.4, 15.0, 14.3. HRMS (APCI^{+}) *m/z*: $[M + H]^{+}$ Calcd for $C_{46}H_{56}BN_6OS_2$: 782.4081; Found 782.4068.

Spectra of 3.

Figure S3. ^{1}H NMR (300 MHz, $CDCl_3$) of **3**

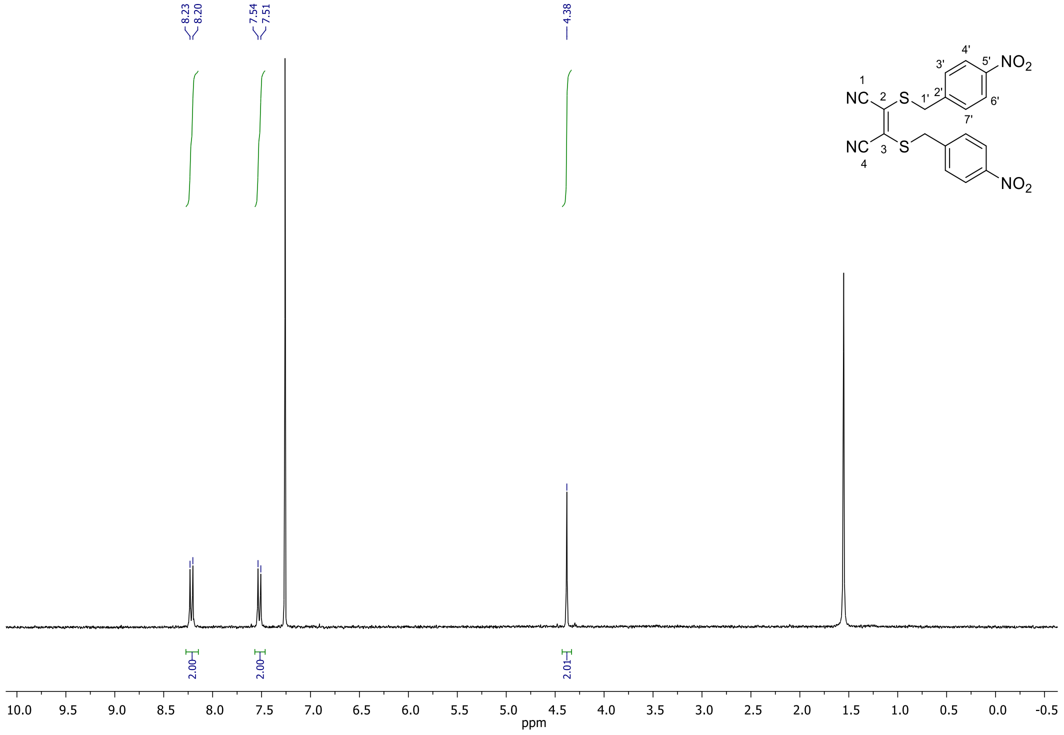

Figure S4. ^{13}C NMR (75.5 MHz, $CDCl_3$) of **3**

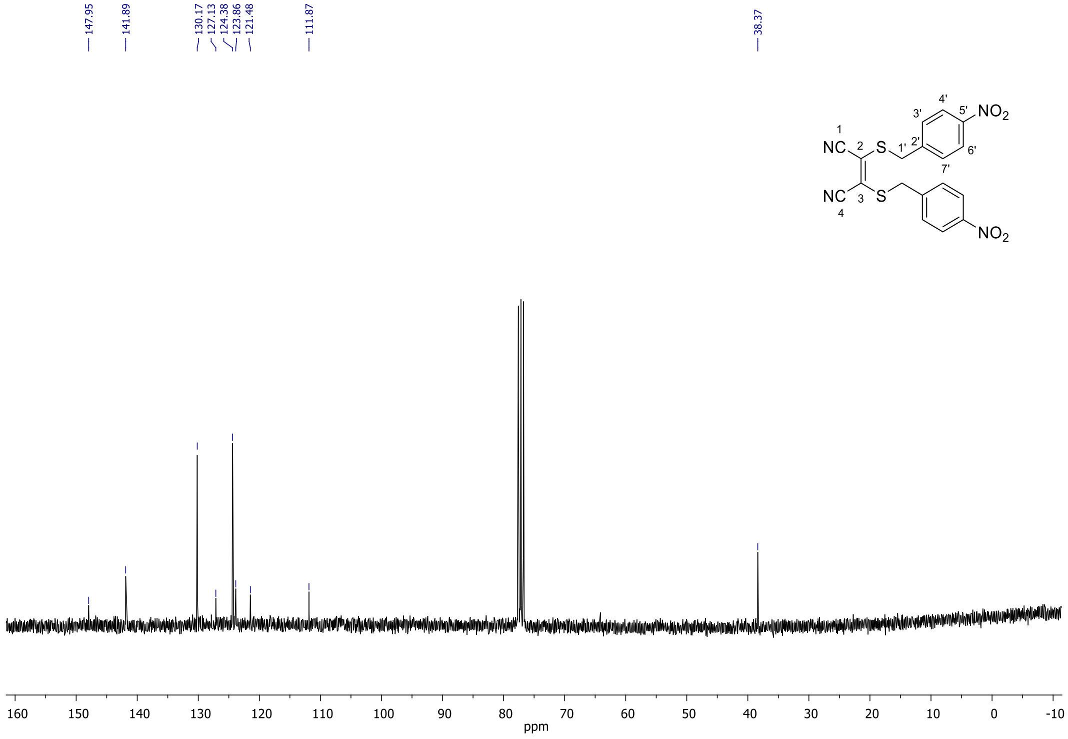

Figure S5. FT-IR (ATR) Spectrum of **3**

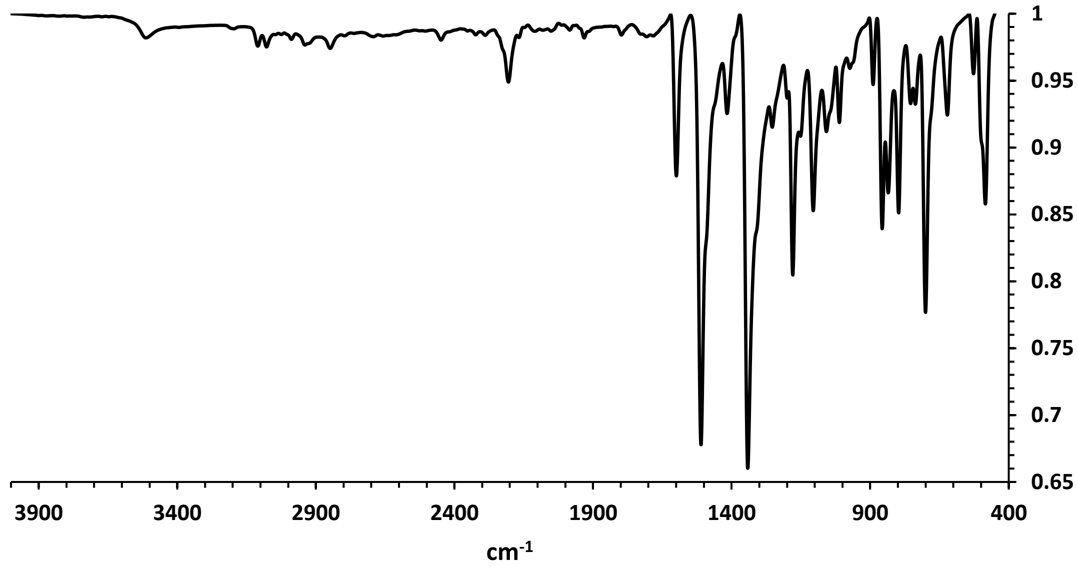

Figure S6. MS (APCI, +) of **3.**

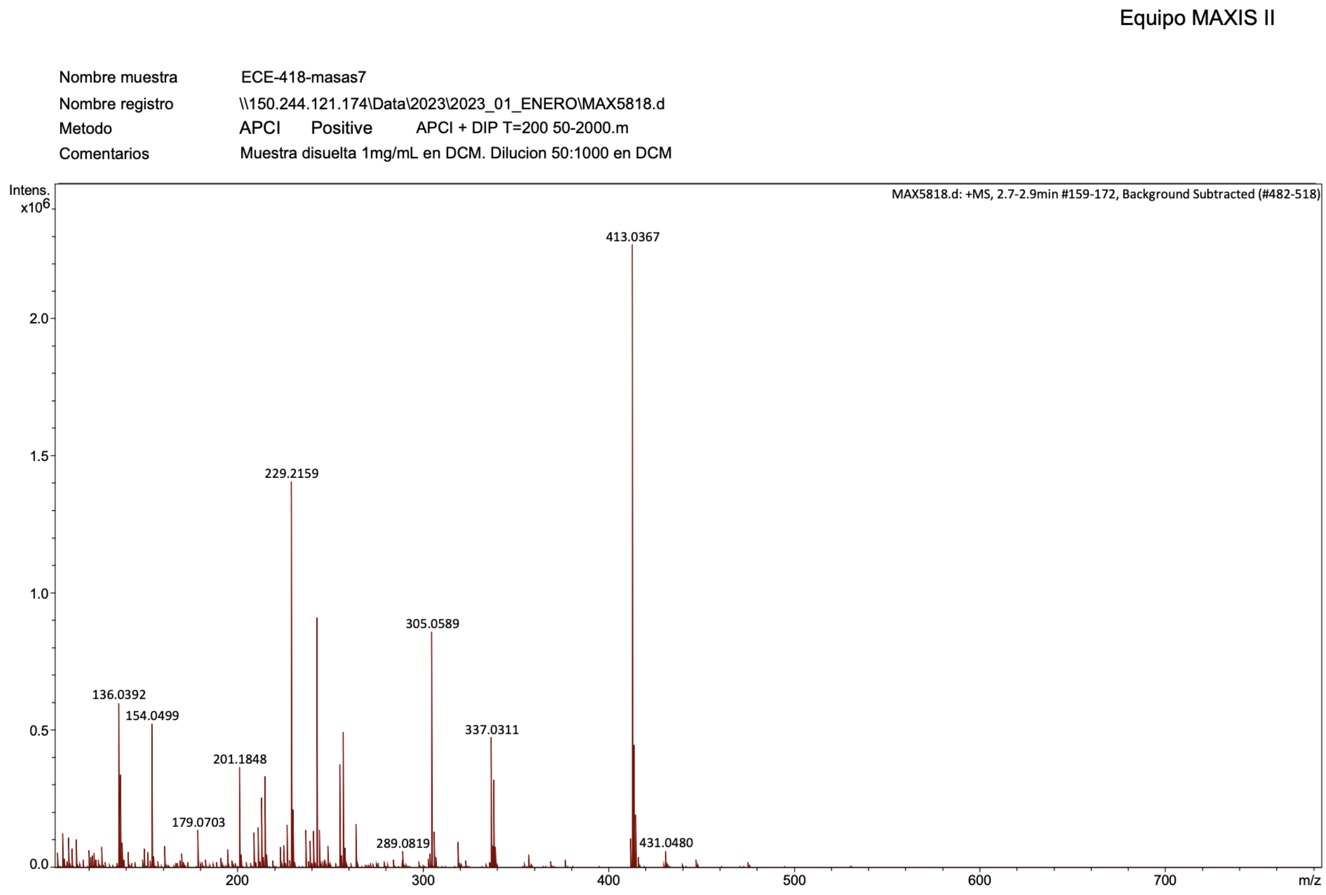

Figure S7. HRMS (APCI, +) of **3**: Upper: found; Lower: calculated

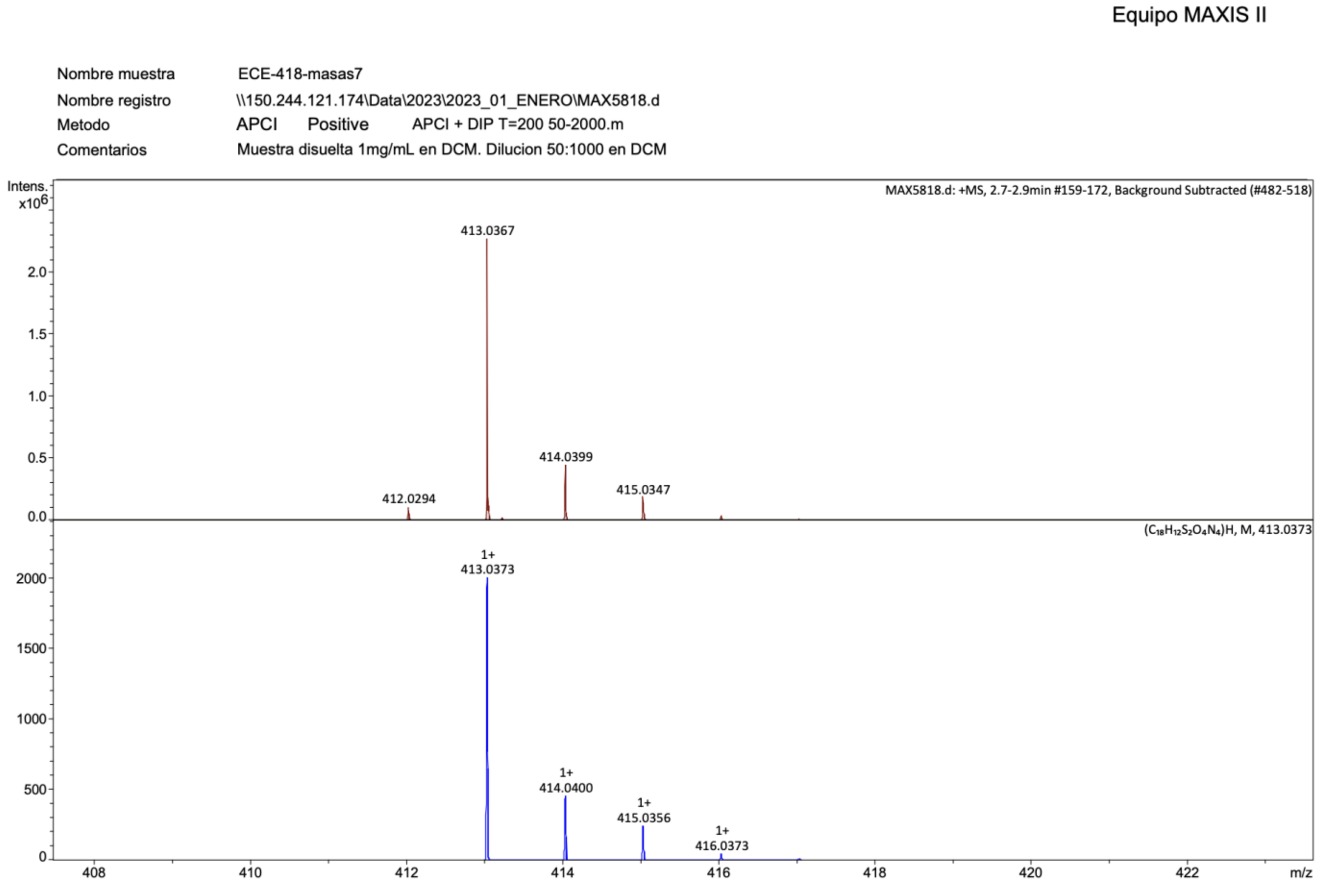

Spectra of 4

Figure S8. ^{1}H NMR (300 MHz, $CDCl_3$) of **4**

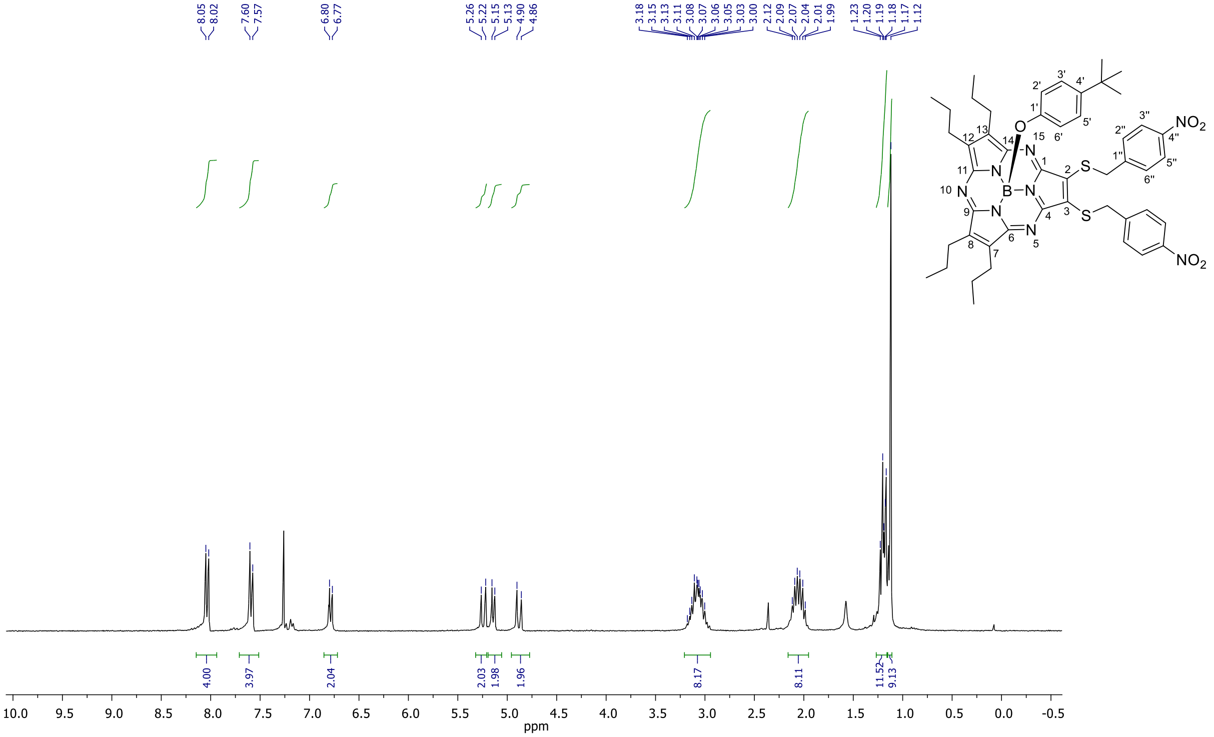

Figure S9. ^{13}C NMR (75.5 MHz, $CDCl_3$) of **4**

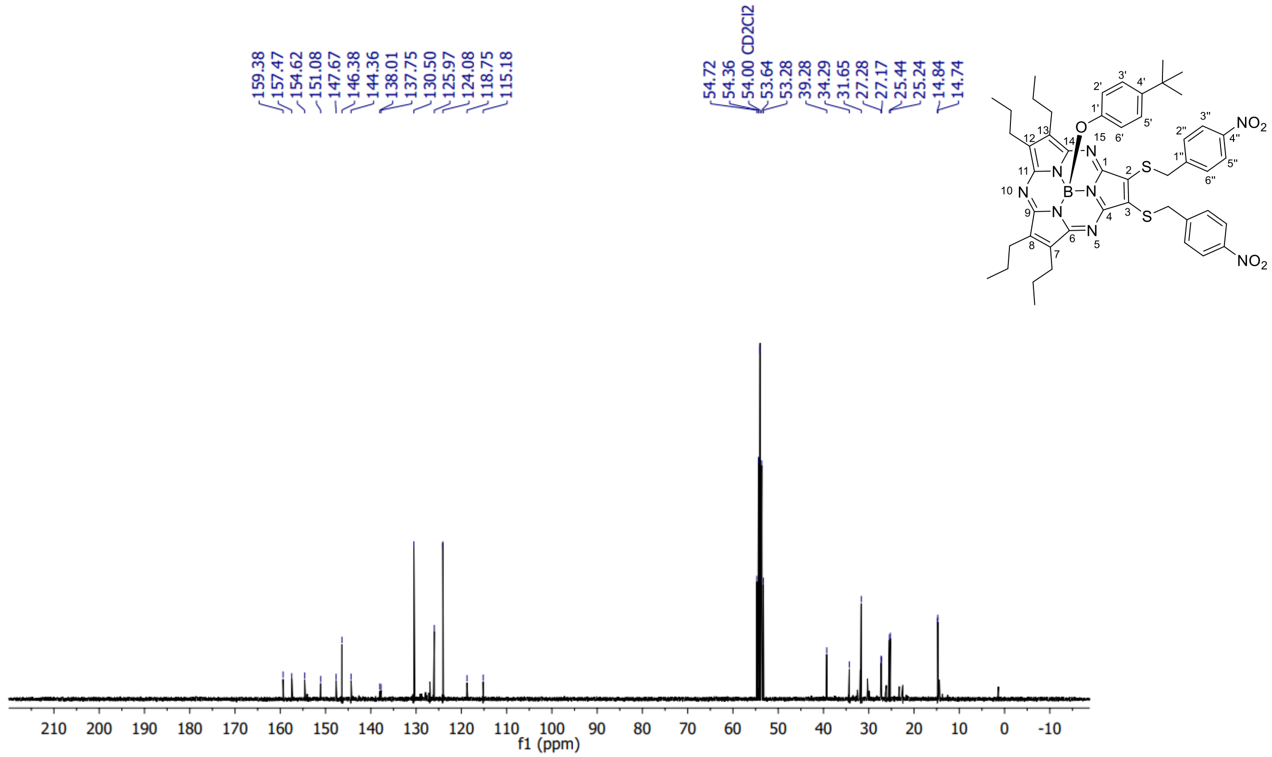

Figure S10. UV-Vis spectrum of **4** in $CHCl_3$

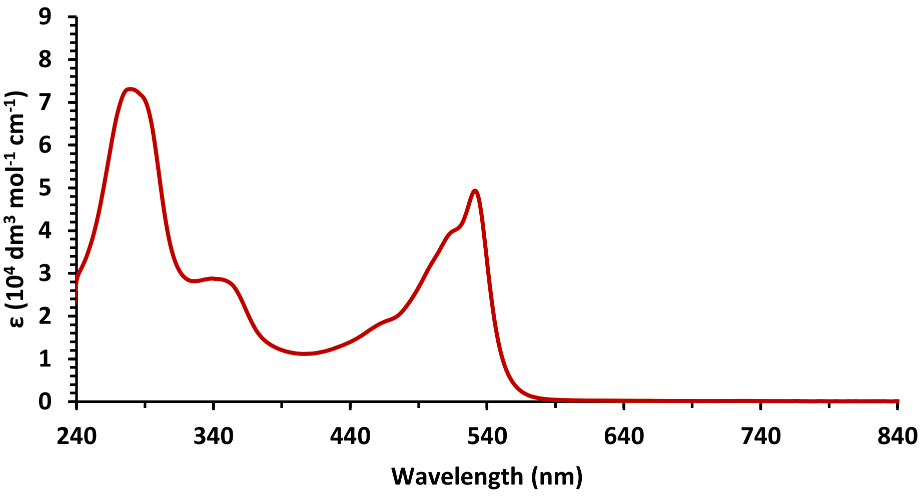

Figure S11. FT-IR (film) of **4**

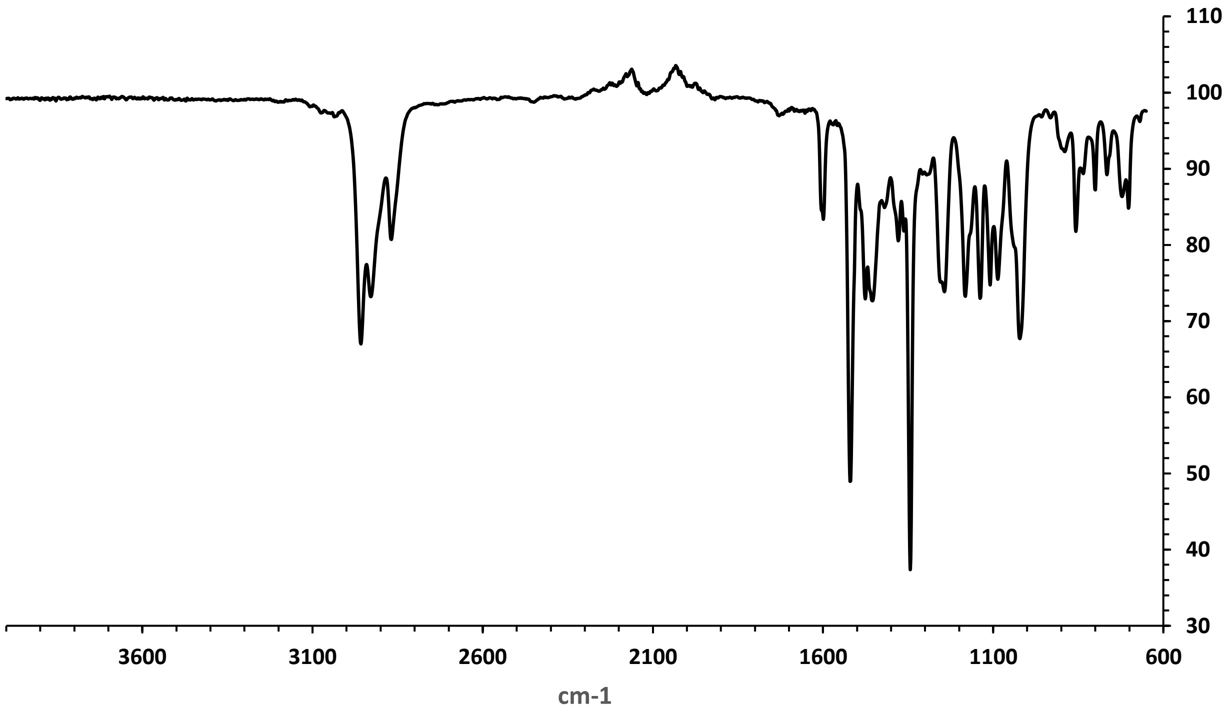

Figure S12. Normalized absorbance (continuous line)and emission (dashed line) spectra of **4** in $CHCl_3$

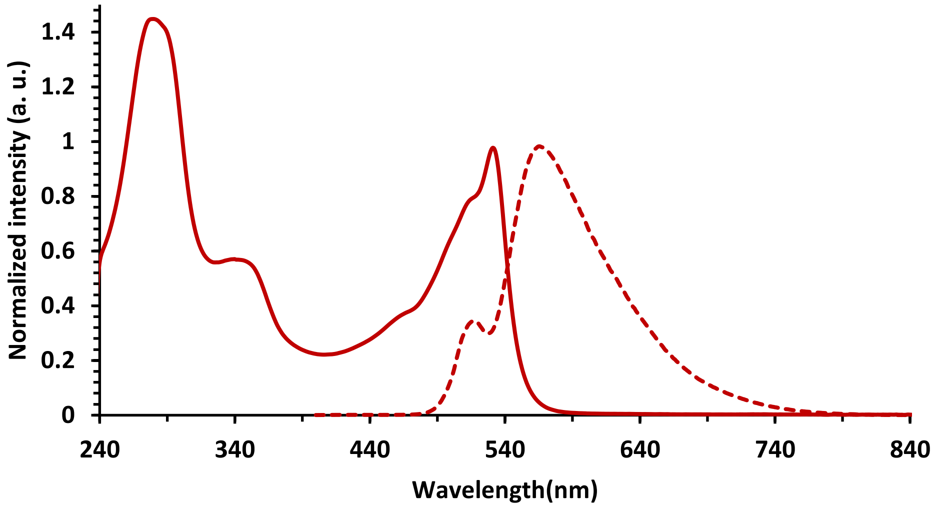

Figure S13. MS (MALDI-TOF$^+$, DCTB+NaI): of **4**

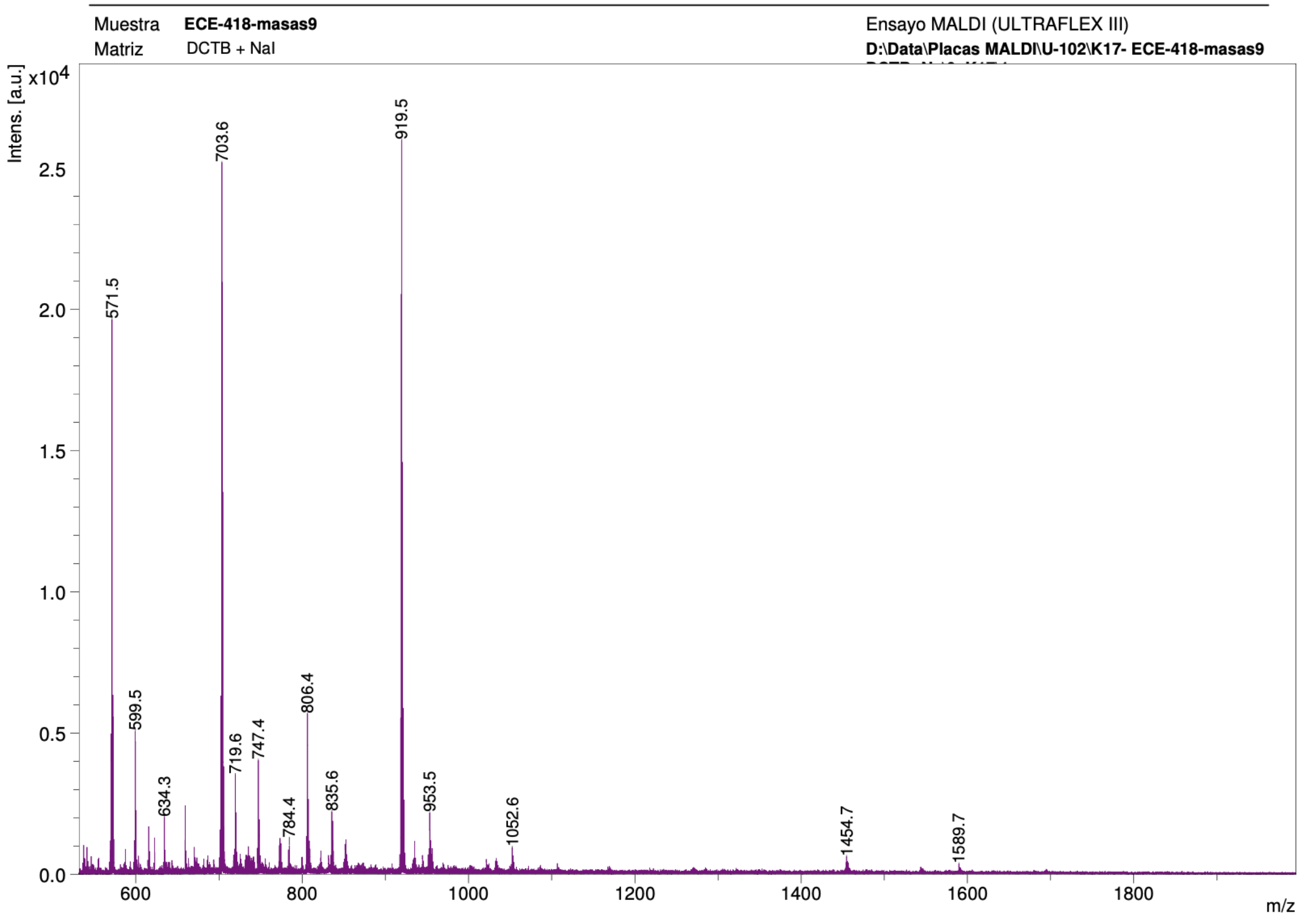

Figure S14. HRMS MALDI-TOF+ (DCTB+PPGNa1000+NaI) of **4** with isotopic pattern (Upper: found; Lower: calculated)

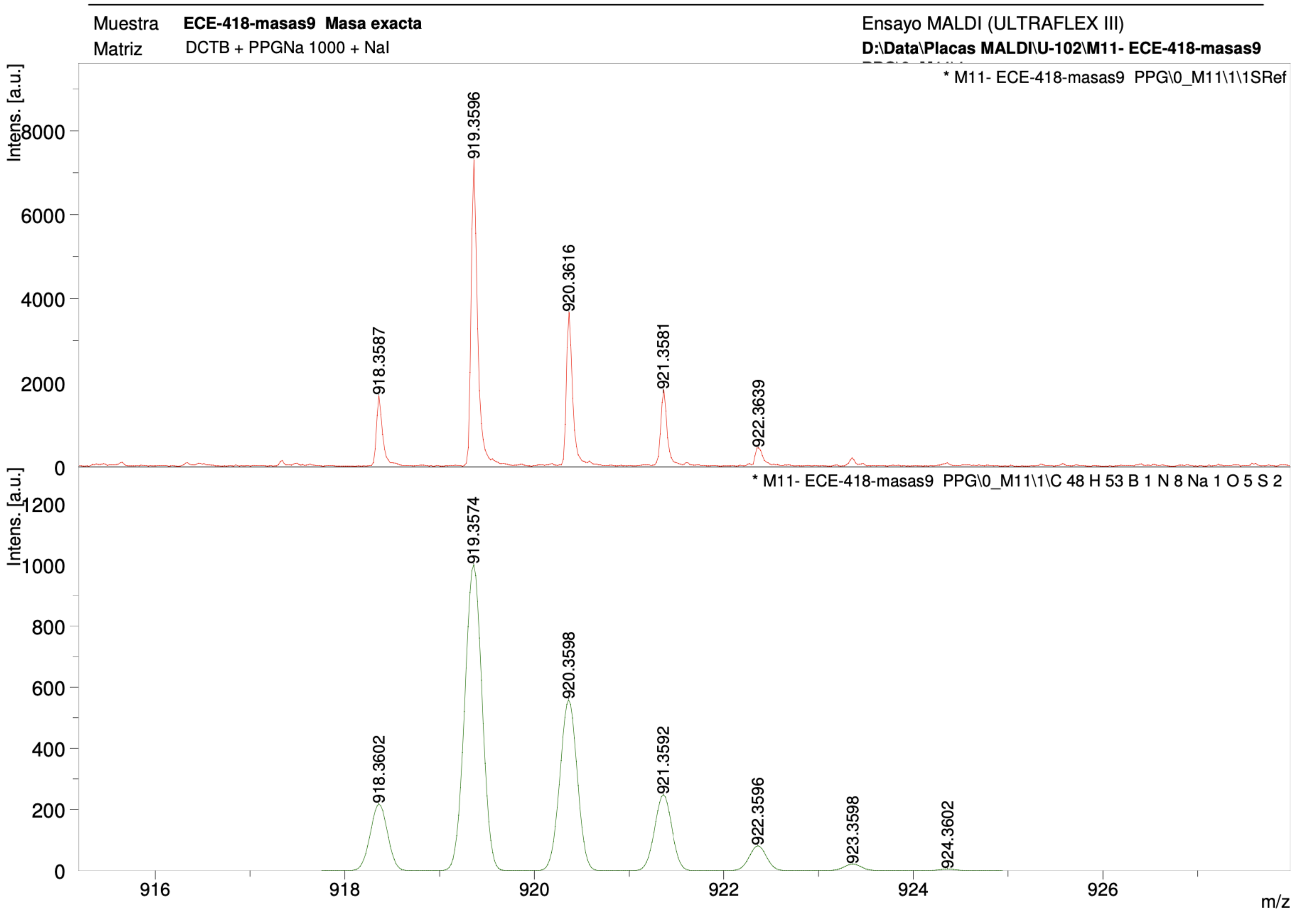

Spectra of SubPz-DTE 1

Spectra of SubPz-DTE 1*o*

Figure S15. ^{1}H NMR (300 MHz, $CDCl_3$) of **1*o***

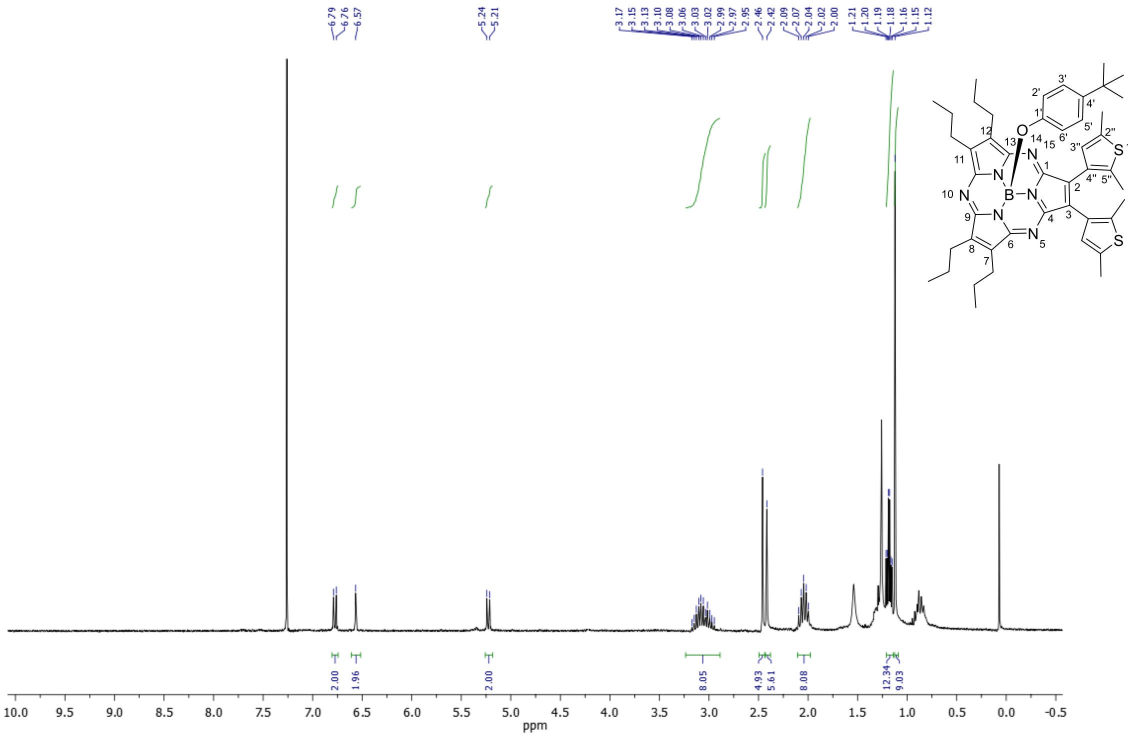

Figure S16. ^{1}H NMR (300 MHz, toluene-d_8) of **1*o***

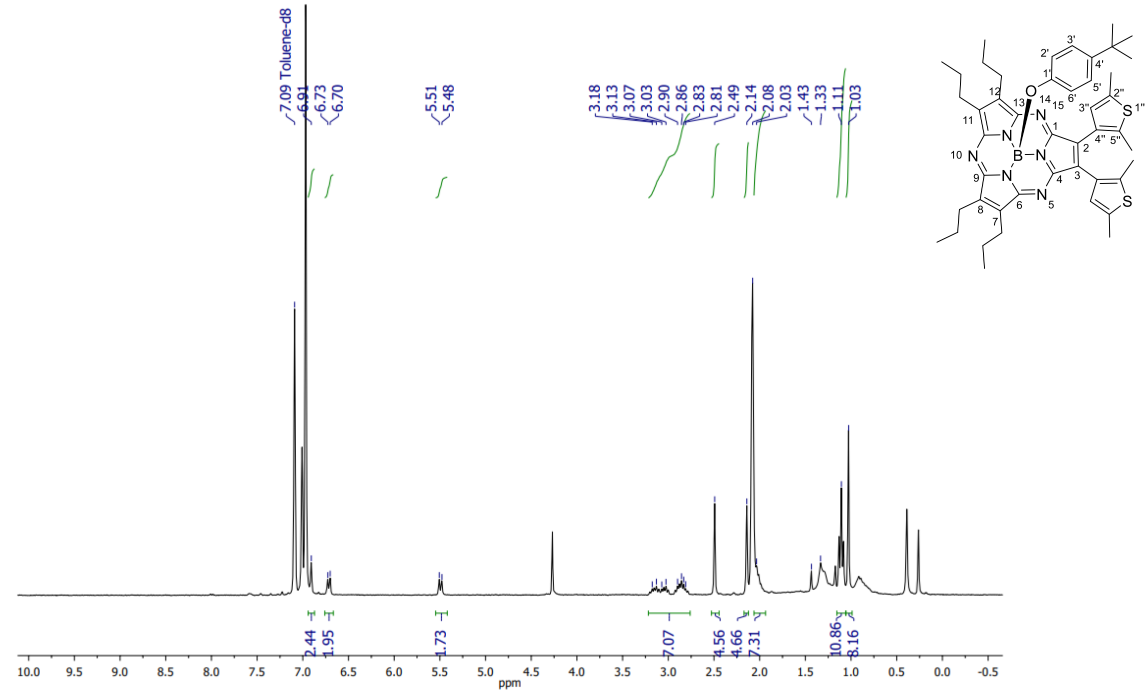

Figure S17. ^{13}C NMR (75.5 MHz, toluene-d$_8$) of **1*o***

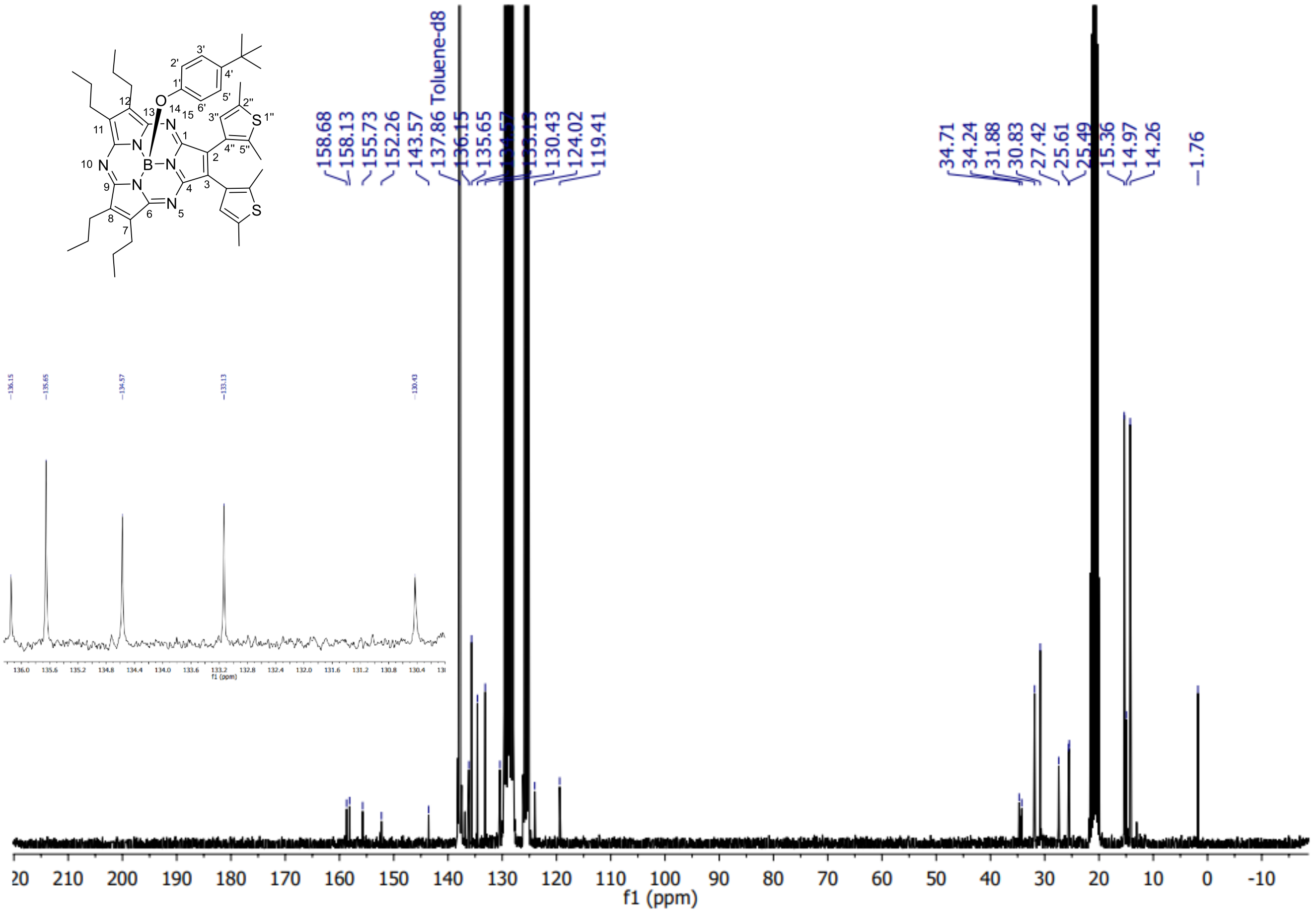

Figure S18. UV-Vis spectrum of **1*o*** in toluene.

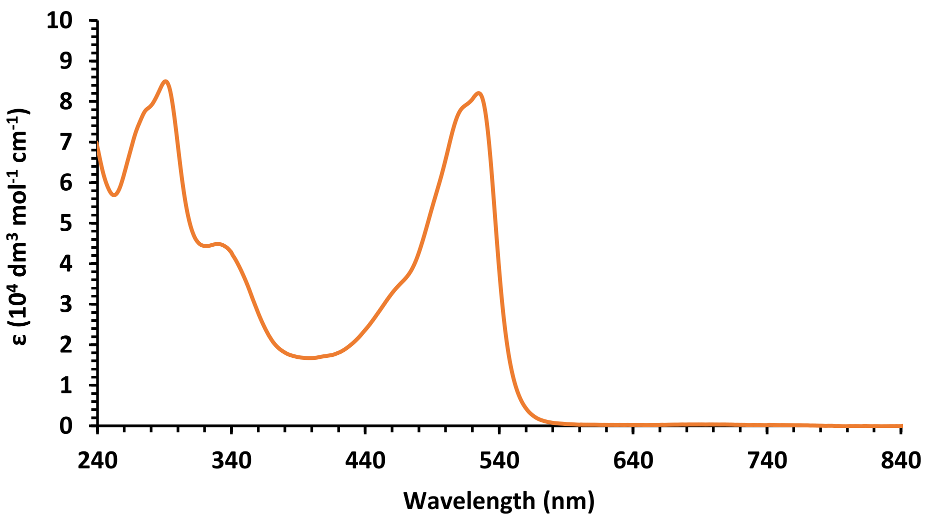

Figure S19. FT-IR (ATR) spectrum of **1*o***

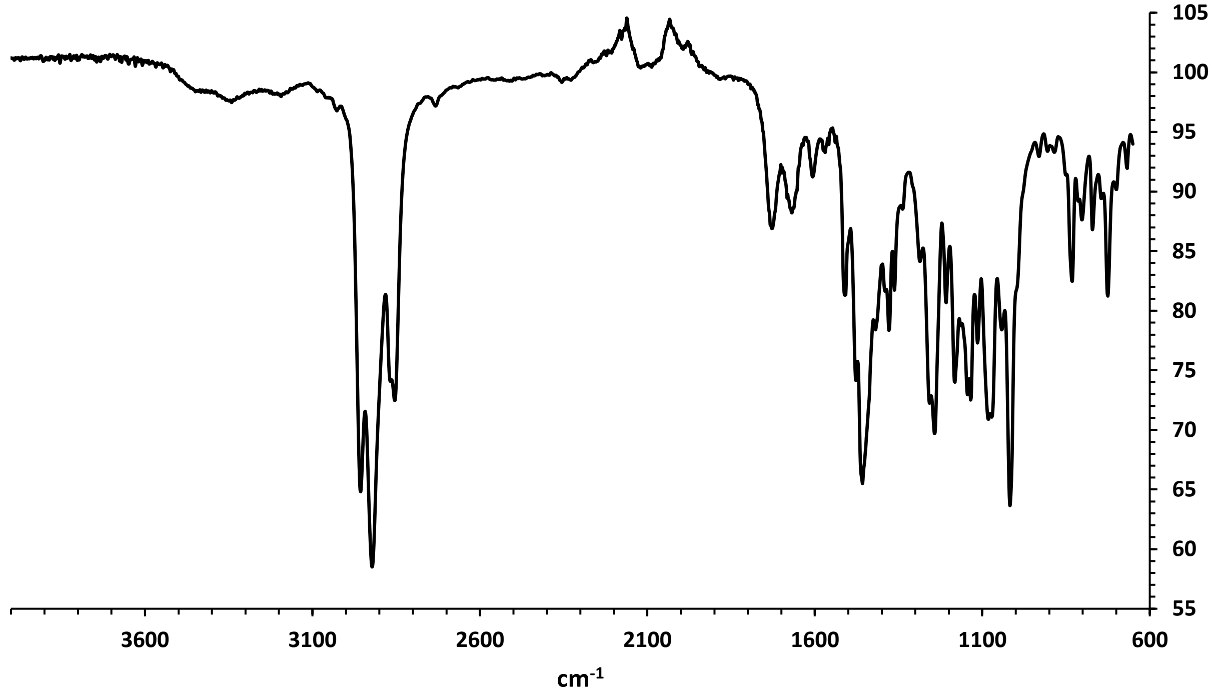

Figure S20. Normalized absorption (solid line) and emission (dashed line) spectrum of **1*o*** in toluene

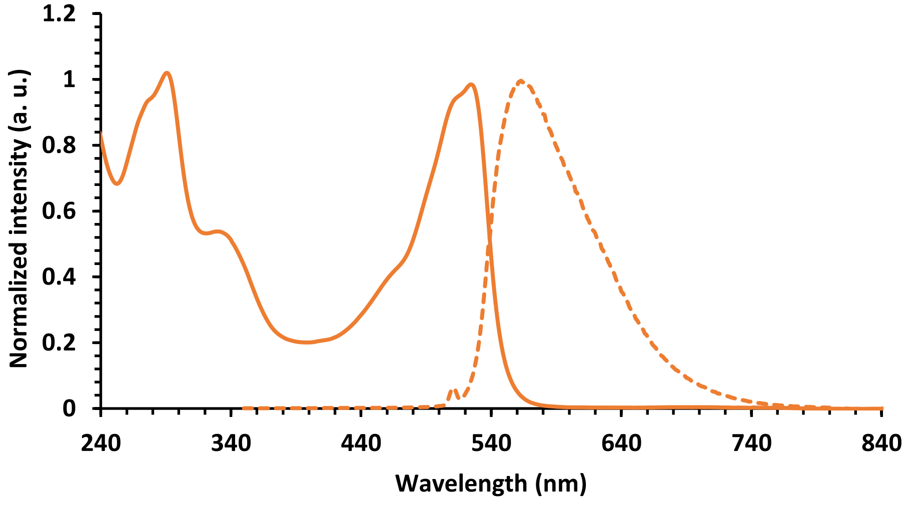

Figure S21. HRMS (APCI$^+$, DCM): of **1*o*** with isotopic pattern: Upper: found; Lower: calculated

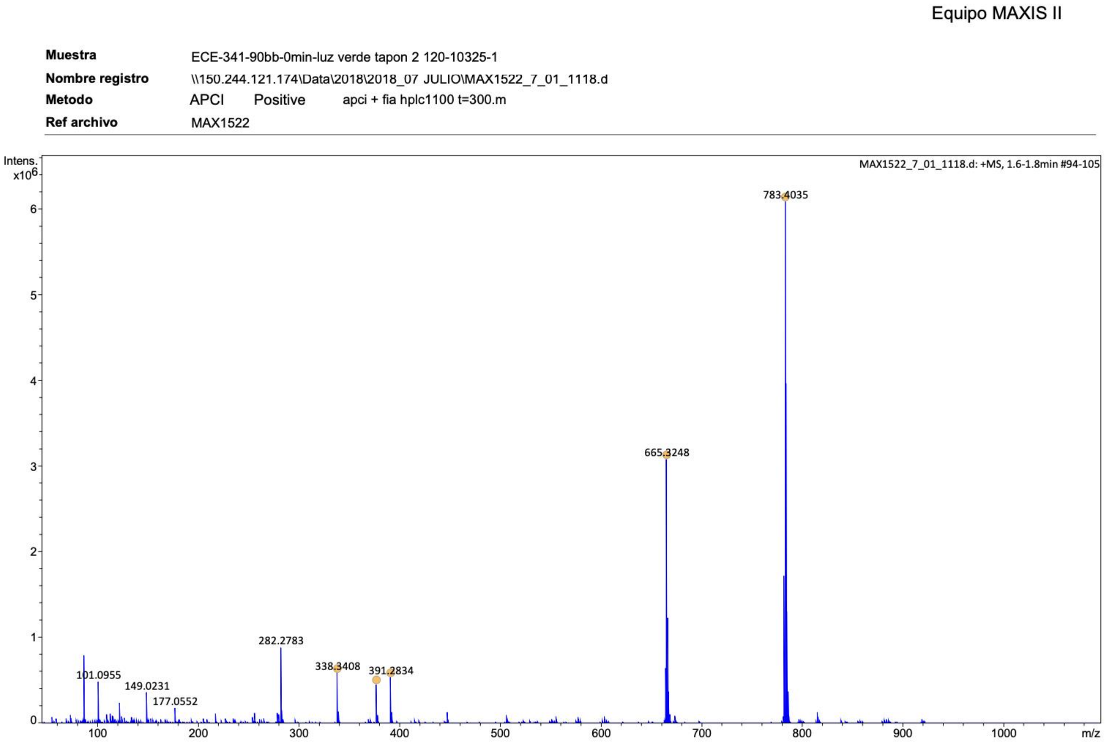

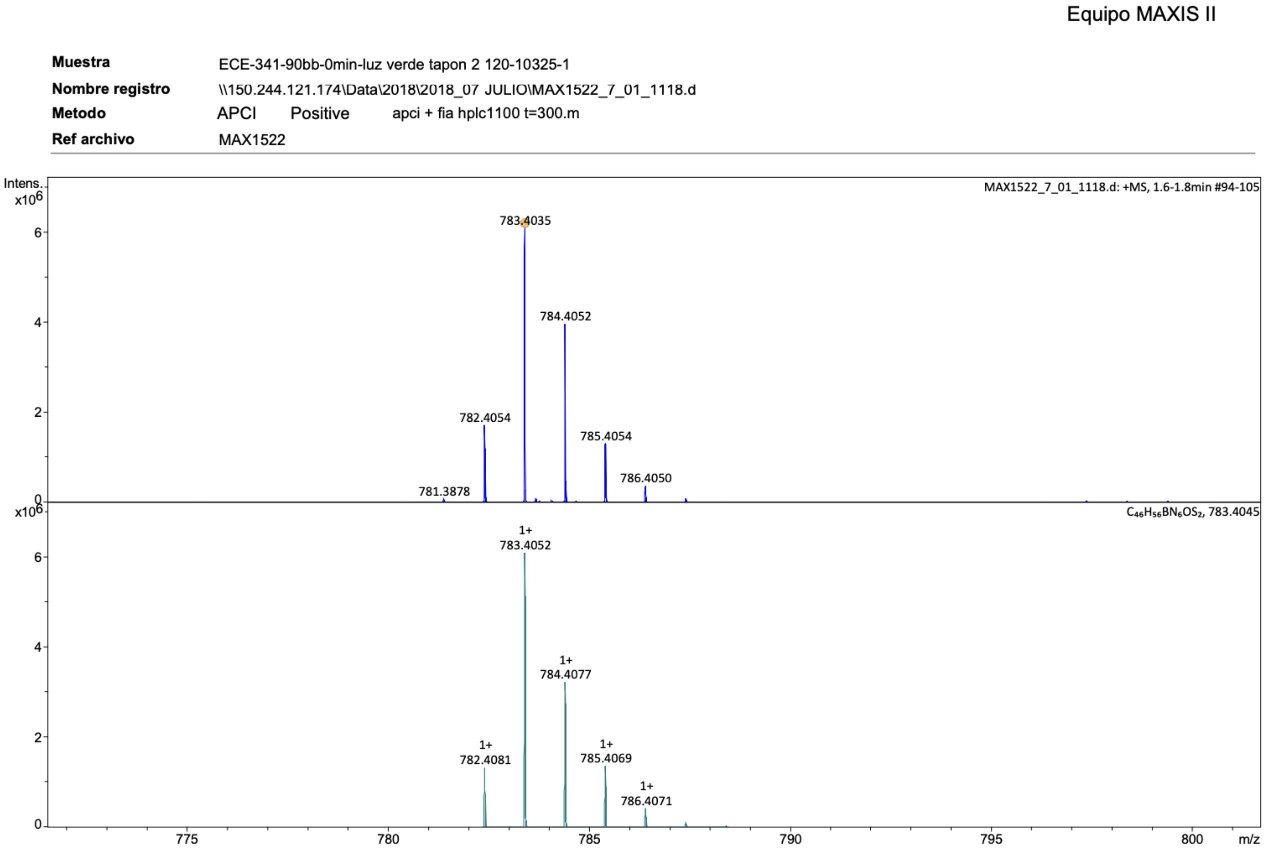

Photoisomerization of SubPz-DTE 1*o* – 1*c*

Figure S22. ^{1}H NMR spectral changes of SubPz-DTE **1** in deuterated toluene upon photoexcitation at 515 nm.

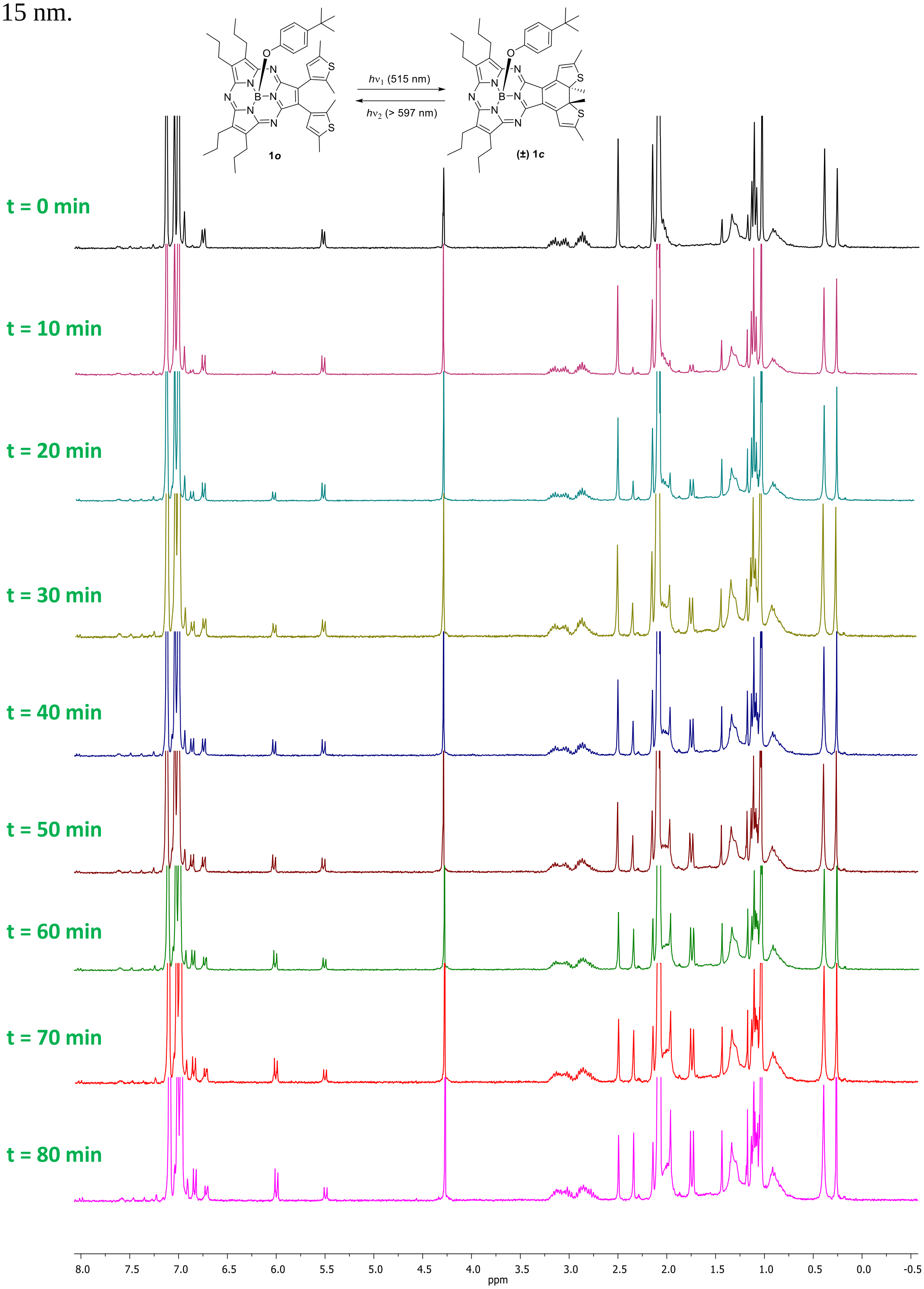

Figure S23. ^{1}H NMR spectral changes of the PSS of SubPz-DTE **1** in deuterated toluene upon photoexcitation at 597 nm.

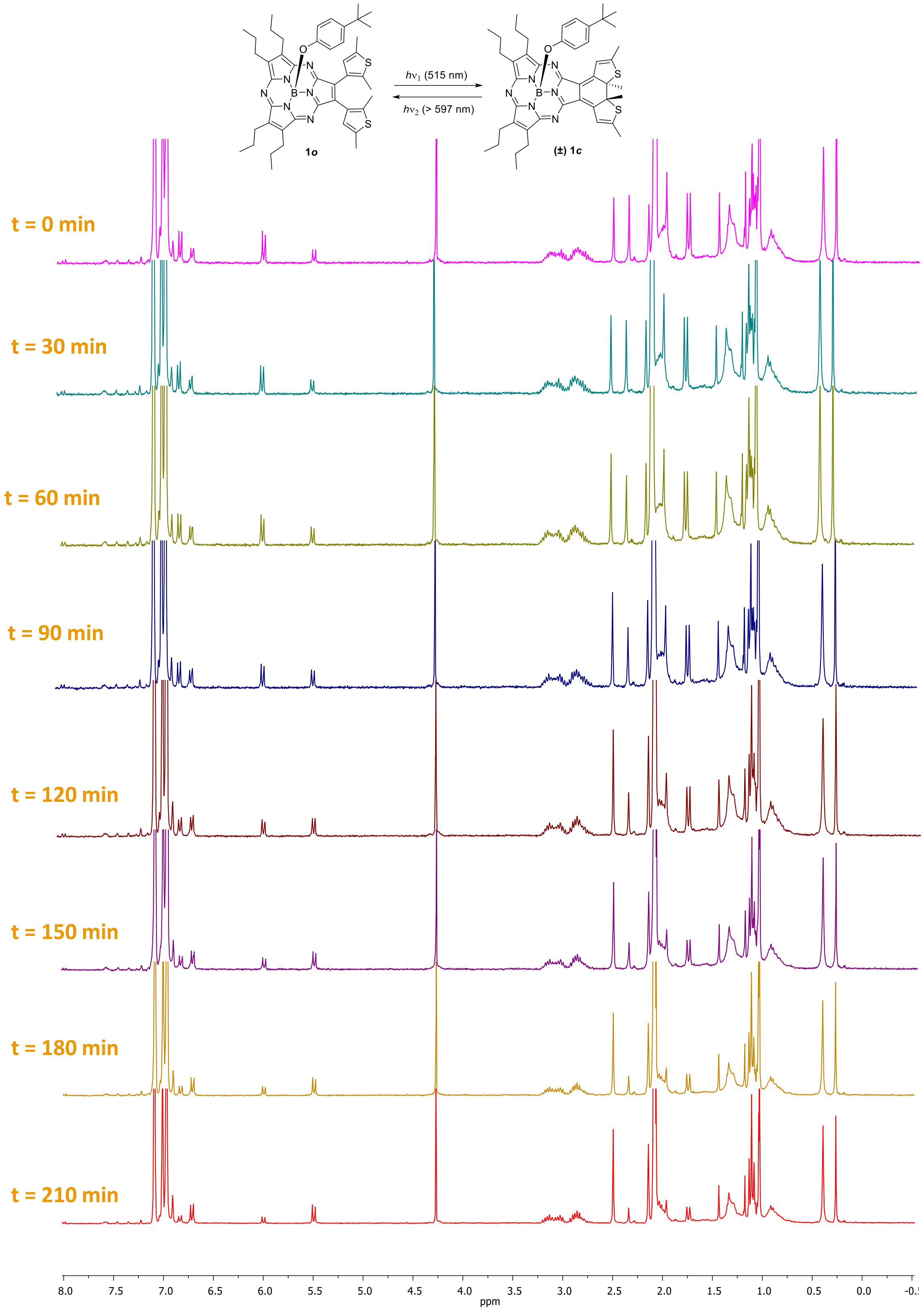

Figure S24. ^{13}C NMR (75.5 MHz, toluene-d_8) of the PSS mixture of SubPz-DTE **1**. Peak peaking includes only the new resonances that appeared with the formation of **1*c*** upon irradiation of **1*o***.

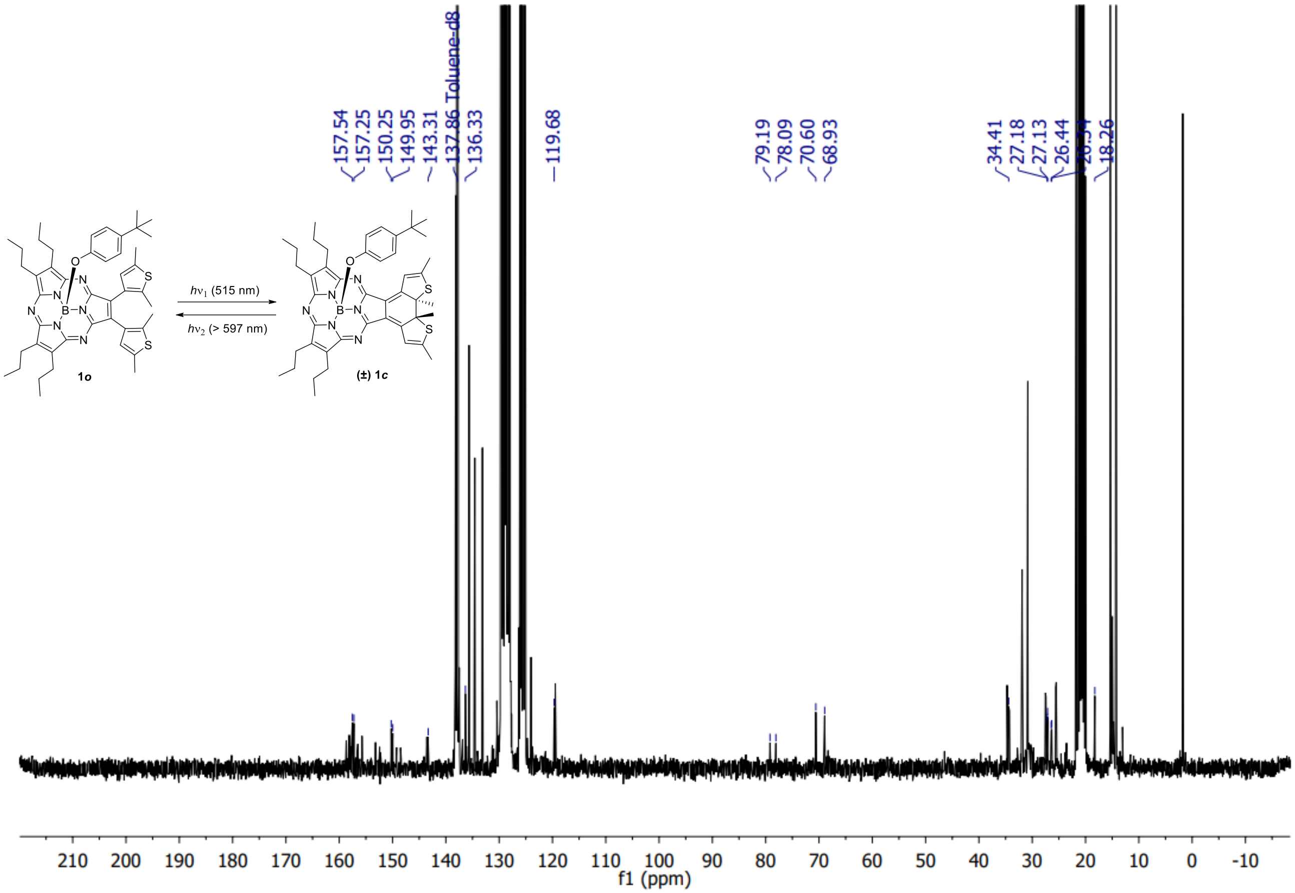

Figure S25. UV-Vis spectrum of **1*c*** estimated by subtracting the initial absorption spectrum of **1*o*** (27% of the initial intensity), from the absorption spectrum of the PSS mixture. Green line: spectrum of **1*o***; Dark red line: spectrum of **1*c***

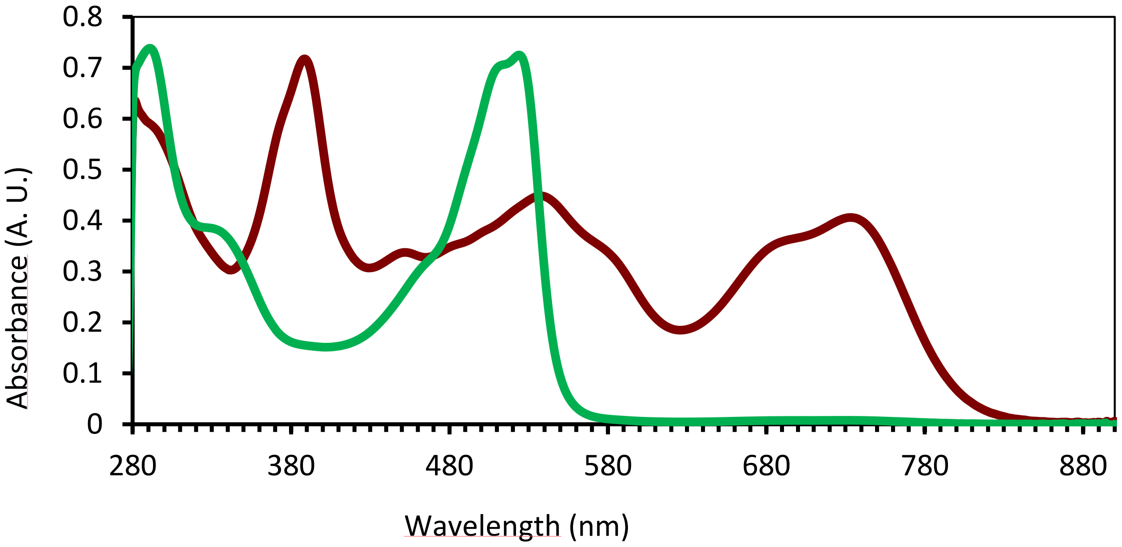

Figure S26. HRMS (APCI$^+$, DCM): of **1*o*** after 80 minutes of irradiation with 515 nm LED light. Isotopic pattern: Upper: found; Lower: calculated

Equipo MAXIS II

**Muestra**	ECE-341-90bb-360 min-luz verde tapon 1 120-10325-2
**Nombre registro**	\\150.244.121.174\Data\2018\2018_07 JULIO\MAX1523_8_01_1119.d
**Metodo**	APCI Positive apci + fia hplc1100 t=300.m
**Ref archivo**	MAX1523

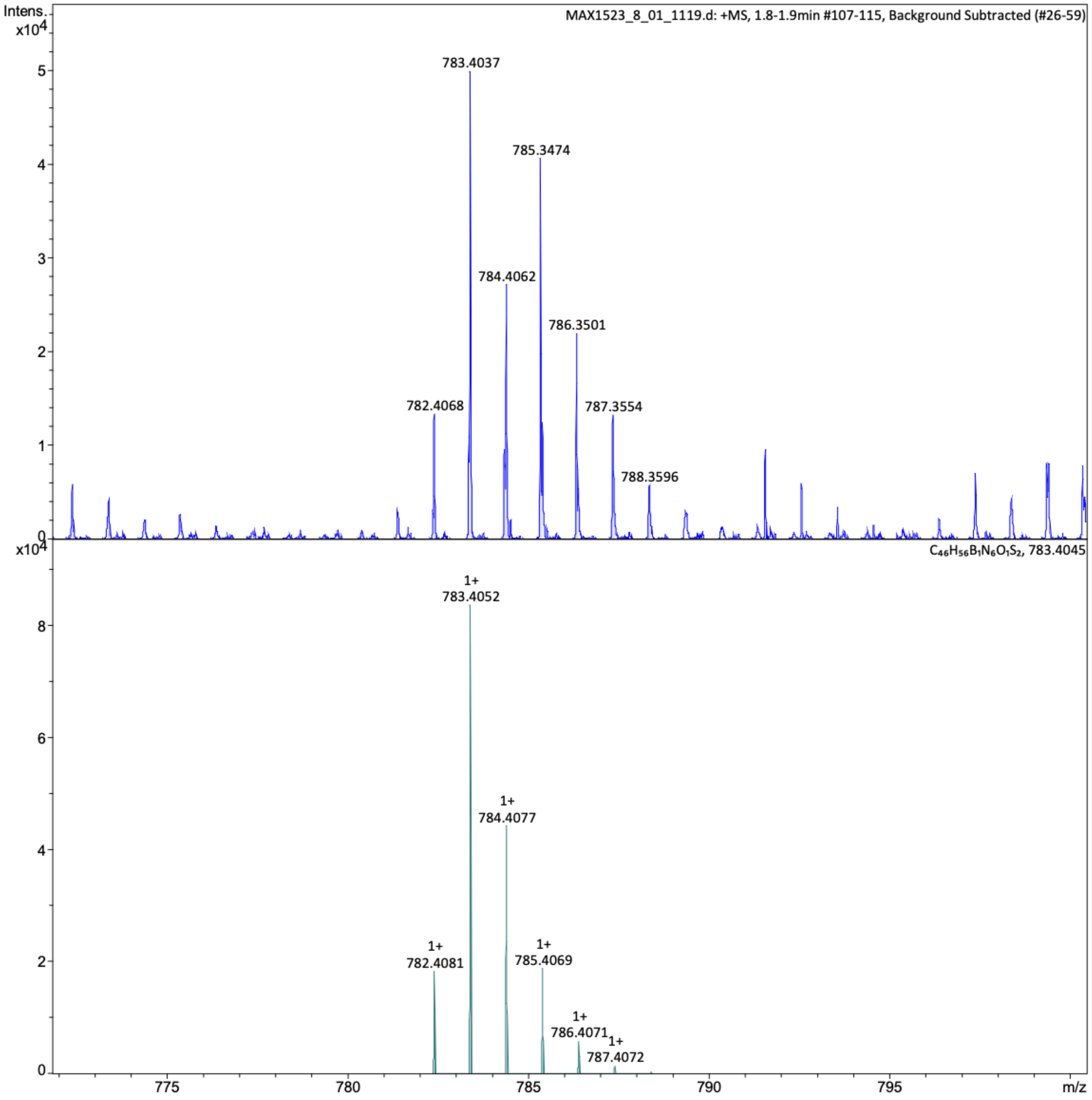

Figure S27. Changes in the UV/vis absorption spectrum of a mixture of **1*o*** and **1*c*** in the PPS ratio in toluene upon heating at 80ºC, monitoring the thermal cycloreversion.

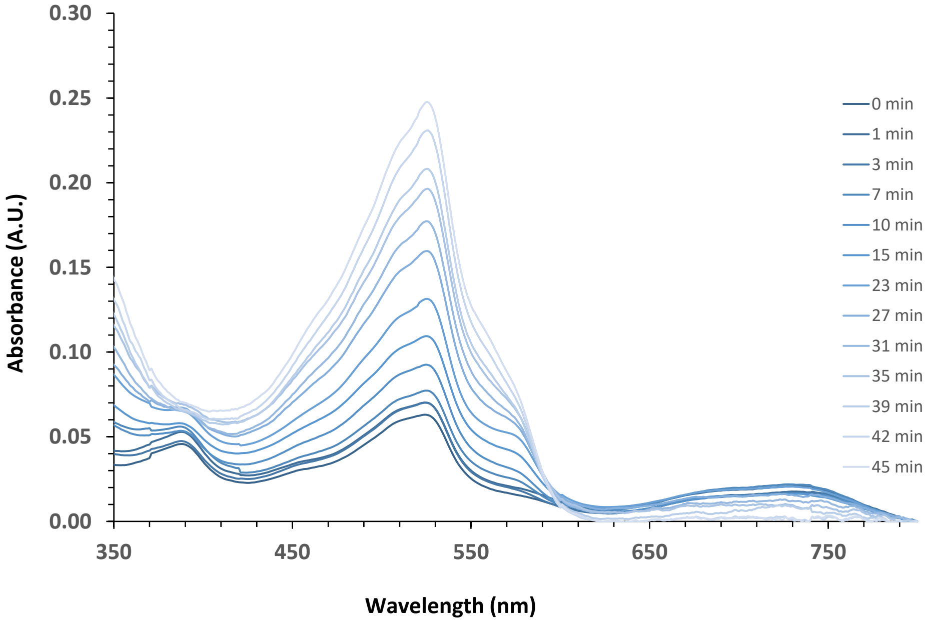

Figure S28. Evolution of absorbance monitored by UV-Vis spectroscopy in a toluene solution of SubPz-DTE **1** during 24 repetitive switching cycles. In the first cycle, **1*o*** is irradiated with 515 nm light for 1.5 min, followed by irradiation with 597 nm light for 6 min. In the subsequent 23 cycles, the solution is irradiated with 515 nm light for 1 min, followed by irradiation with 597 nm light for 6 min. The last irradiation with 597 nm light was for 30 min, until no more photoisomerization was observed. Blue line: Spectrum of **1** before irradiation; Red line: Spectrum of **1** after the 24 switching cycles.

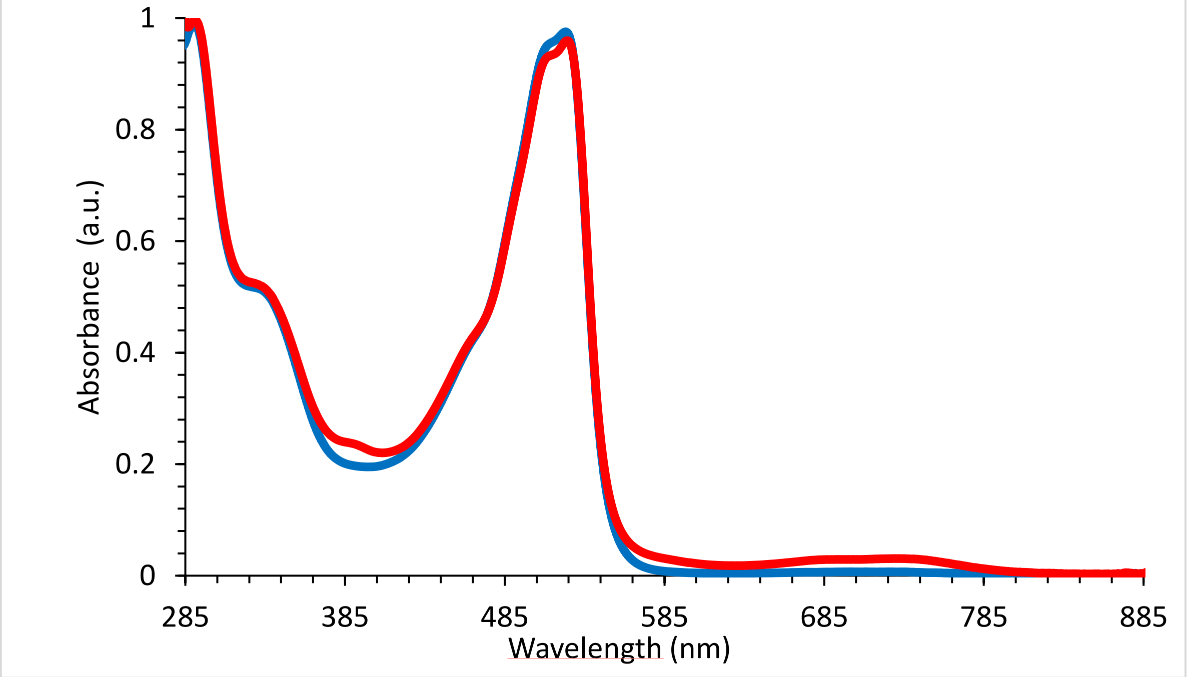

Figure S29. DFT predicted HOMO and LUMO orbitals and frontier orbital energy levels (eV) for a) **1*o*** and b) **1*c***.

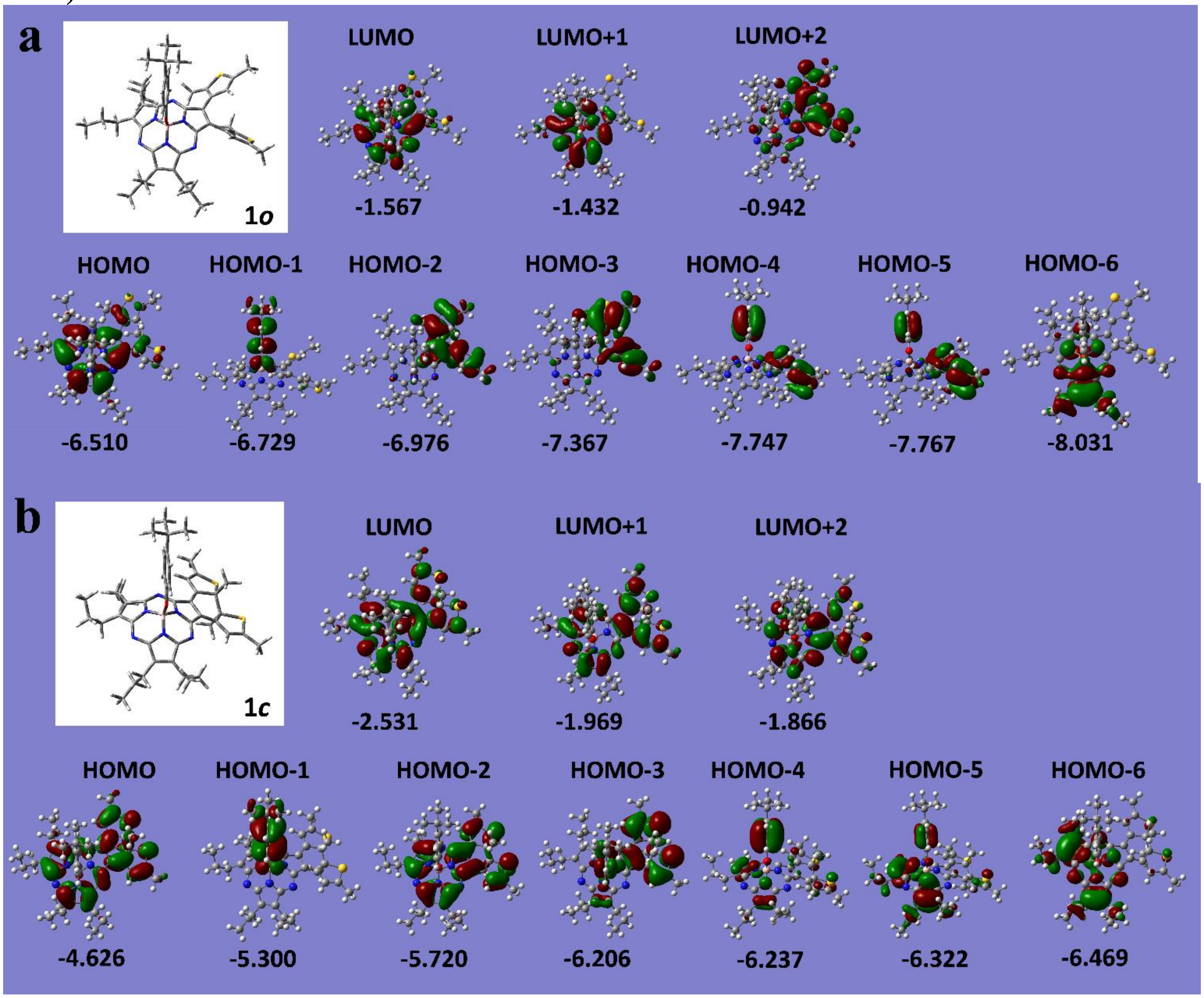

Figure S30. TD-DFT predicted absorption spectra for a) **1*o*** and b) **1*c***.

a) b)

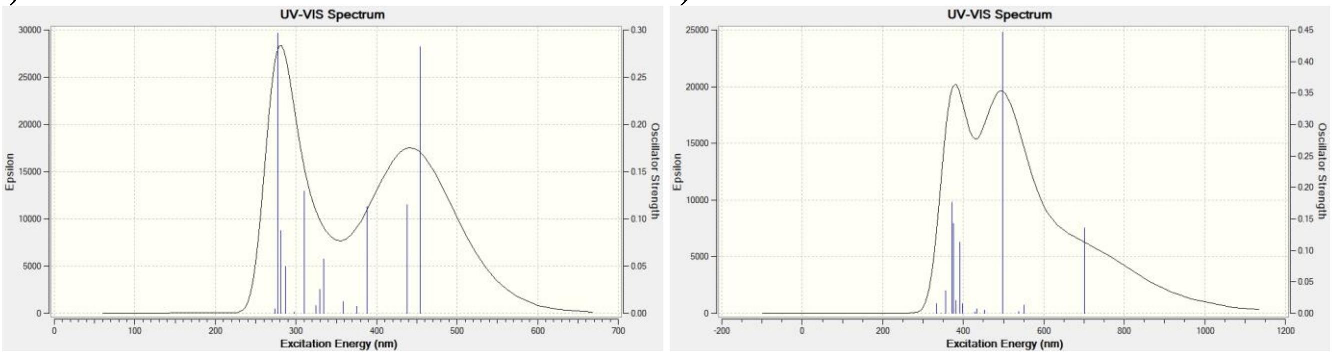

Table S1. Predicted and experimental absorption bands (λ_{max}), Singlet Excited States, oscillator strengths (*f*) and dominant molecular orbital compositions for SubPz-DTE **1*o*** and **1*c***. H and L stands for HOMO and LUMO, respectively.

	Absorption	ES	Predicted λ_{max} (nm)	*f*[a]	Main contributions[b]	Experimental λ_{max} (nm)
**1o**	B bands	S14	277	0.23	H–7 → L (26%)<br>H–6 → L (28%)<br>H–5 → L (30%)<br>H–3 → L+1 (36%)	291
		S13	278	0.30	H–7 → L (28%)<br>H–7 → L+1 (40%)<br>H–6 → L (22%)<br>H–6 → L+1 (27%)	
		S9	310	0.13	H–7 → L (27%)<br>H–6 → L (33%)<br>H–6 → L+1 (22%)<br>H–3 → L (24%)<br>H–2 → L+1 (39%)	329
		S3	388	0.12	H–2 → L (60%)<br>H–1 → L (24%)	463
	Q bands	S2	438	0.11	H → L+1 (65%)	508
		S1	454	0.28	H → L (67%)	527
**1c**	B bands	S12	372	0.18	H–6 → L (51%)<br>H–3 → L (23%)<br>H–2 → L+2 (40%)	388
		S11	376	0.14	H–5 → L (22%)<br>H–2 → L+1 (62%)	
		S9	392	0.11	H–3 → L (61%)<br>H–2 → L+2 (25%)	452
	Q bands	S4	497	0.45	H → L+2 (69%)	538
		S1	702	0.14	H → L (70%)	740

[a]Only transitions with $f \geq 0.09$ are reported.
[b]The most important contributions (> 20%) are reported.

Figure S31. Photodecomposition of DPBF by $^1O_2$ in DMSO after irradiation of **1*o*** monitoring the maximum absorption of DPBF at 417 nm. Inset: Time dependent photodegradation of DPBF.

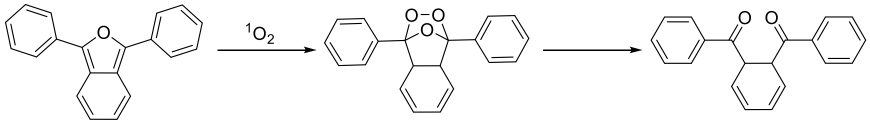

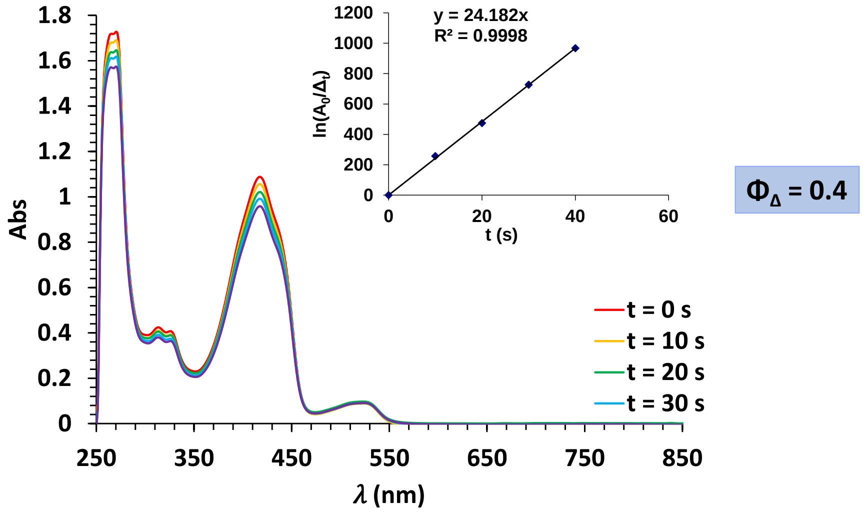

Figure S32. Photodecomposition of DPBF by $^1O_2$ in DMSO after irradiation of the mixture of **1*o*** and **1*c*** in the PSS ratio monitoring the maximum absorption of DPBF at 417 nm. Inset: Time dependent photodegradation of DPBF.

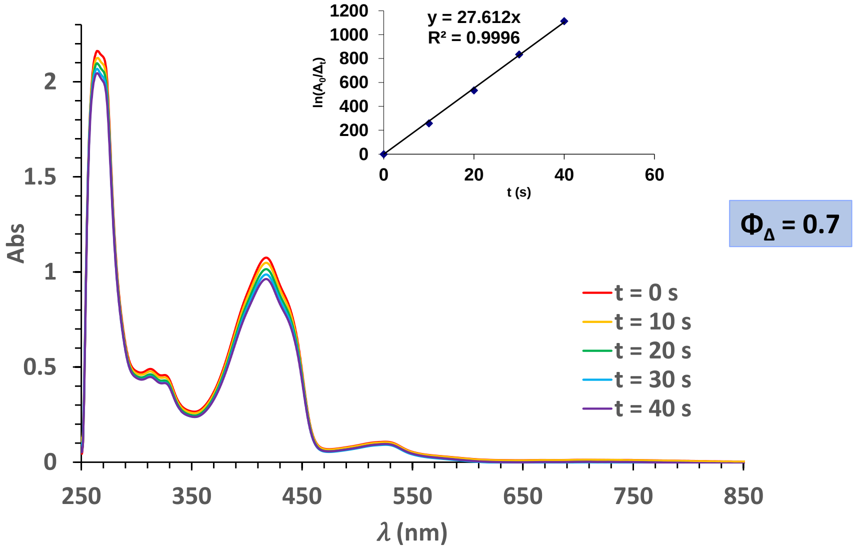

References

[1] Davison, A.; Holm, R. H.; Benson, R. E.; Mahler, W. Metal Complexes Derived from cis-1,2-dicyano-1,2-ethylenedithiolate and Bis(Trifluoromethyl)-1,2-dithiete, *Inorganic Syntheses* **1967**, *10*, 8-26.

[2] Lange, S. J.; Nie, H.; Stern, C. L.; Barrett, A. G. M.; Hoffman, B. M. Peripheral Palladium(II) and Platinum(II) Complexes of Bis(dimethylamino)porphyrazine, *Inorg. Chem.* **1998**, *37*, 6435-6443.

[3] (a) Ko, C.; Kwok, W.; Yam, V. W.; Phillips, D. L. Triplet MLCT Photosensitization of the Ring-Closing Reaction of Diarylethenes by Design and Synthesis of a Photochromic Rhenium(I) Complex of a Diarylethene-Containing 1,10-Phenanthroline Ligand *Chem. Eur. J.* **2006**, *12*, 5840-5848. (b) Seon-Jeong, L.; Byeong-Kwan, A.; Soo-Young, P. Bistable Photoswitching in the Film of Fluorescent Photochromic Polymer: Enhanced Fluorescence Emission and Its High Contrast Switching, *Macromolecules* **2005**, *38*, 6236-6239.

[4] Higashino, T.; Rodríguez-Morgade, M. S.; Osuka, A.; Torres, T. Peripheral Arylation of Subporphyrazines. *Chem. Eur. J.* **2013**, *19*, 10353-10359.

[5] Maree, M. D.; Kuznetsova, N.; Nyokong, T. Silicon octaphenoxyphthalocyanines: photostability and singlet oxygen quantum yields, *J. Photochem. Photobiol. A Chem.* **2001**, *140*, 117-125.

[6] Tau, P.; Ogunsipe, A. O.; Maree, S.; Maree, M. D.; Nyokong, T. Influence of cyclodextrins on the fluorescence, photostability and singlet oxygen quantum yields of zinc phthalocyanine and naphthalocyanine complexes, *J. Porphyrins Phthalocyanines* **2003**, *7*, 439-446.

[7] Frisch, M.J.; Trucks, G.W.; Schlegel, H.B.; Scuseria, G.E.; Robb, M.A.; Cheeseman, J.R.; Scalmani, G.; Barone, V.; Petersson, G.A.; Nakatsuji, H.; Li, X.; Caricato, M.; Marenich, A.V.; Bloino, J.; Janesko, B.G.; Gomperts, R.; Mennucci, B.; Hratchian, H.P.; Ortiz, J.V.; Izmaylov, A.F.; Sonnenberg, J.L.; Williams-Young, D.; Ding, F.; Lipparini, F.; Egidi, F.; Goings, J.; Peng, B.; Petrone, A.; Henderson, T.; Ranasinghe, D.; Zakrzewski, V.G.; Gao, J.; Rega, N.; Zheng, G.; Liang, W.; Hada, M.; Ehara, M.; Toyota, K.; Fukuda, R.; Hasegawa, J.; Ishida, M.; Nakajima, T.; Honda, Y.; Kitao, O.; Nakai, H.; Vreven, T.; Throssell, K.; Montgomery, J. A., Jr.; Peralta, J.E.; Ogliaro, F.; Bearpark, M.J.; Heyd, J. J.; Brothers, E.N.; Kudin, K.N.; Staroverov, V.N.; Keith, T.A.; Kobayashi, R.; Normand, J.; Raghavachari, K.; Rendell, A.P.; Burant, J.C.; Iyengar, S.S.; Tomasi, J.; Cossi, M.; Millam, J.M.; Klene, M.; Adamo, C.; Cammi, R.; Ochterski, J.W.; Martin, R.L.; Morokuma, K.; Farkas, O.; Foresman, J.B.; Fox, D.J. Gaussian, Inc., Wallingford CT, 2016.

[8] Lamsabhi, A. M.; Yáñez, M.; Mó, O.; Trujillo, C.; Blanco, F.; Alkorta, I.; Elguero, J.; Caballero, E.; Rodríguez-Morgade, M. S.; Claessens, C. G. TDDFT study of the UV-vis spectra of subporphyrazines and subphthalocyanines, *J. Porphyrins Phthalocyanines* **2011**, *15*, 1220-1230.
